# Effects of sustained viral response on lipid in Hepatitis C: a systematic review and meta-analysis

**DOI:** 10.1186/s12944-023-01957-2

**Published:** 2024-03-09

**Authors:** Tingting Mei, Xiaojie Huang, Shan Tang, Menglu Liu, Wenyan Zhang, Haibin Yu

**Affiliations:** 1 Interventional Therapy Center for Oncology Beijing Youan Hospital, Capital Medical University, Beijing, 100069 China; 2 Center for Infectious Diseases, Beijing Youan Hospital, Capital Medical University, Beijing, 100069 China; 3 Department of Liver Disease, Beijing Youan Hospital, Capital Medical University, Beijing, 100069 China; 4 State Clinical Drug Trial Institution, Beijing Youan Hospital, Capital Medical University, Beijing, 100069 China

**Keywords:** Direct-acting Antiviral agents, Hepatitis C, Sustained viral response, Lipid, Meta-analysis

## Abstract

**Background:**

Direct-acting Antiviral Agents (DAAs) influence serum lipids of patients with Hepatitis C virus (HCV). This paper presents an analysis of the relevant literature to investigate the effects of DAAs in treating hepatitis C to achieve a sustained viral response (SVR) on lipid parameters.

**Methods:**

PubMed,Web of science, Embase and Central databases were searched, with a deadline of September 2023. Studies on the effects of sustained viral response on lipid parameters after DAAs treatment for hepatitis C were selected. The required information was extracted from the included studies, and then the Stata 12.0 was used to analyze the data quantitatively.

**Results:**

Of 32 studies, the results showed that total cholesterol (TC) levels increased from the end of treatment (WMD = 20.144, 95%CI = 3.404, 36.884,*P* = 0.018) to one year after treatment (WMD = 24.900, 95%CI = 13.669, 36.131, *P* < 0.001). From the end of treatment (WMD = 17.728, 95%CI = 4.375, 31.082, *P* = 0.009) to one year after treatment (WMD = 18.528, 95%CI = 7.622, 29.433, *P* < 0.001), the levels of low-density lipoprotein (LDL) were also increased. High-density lipoprotein (HDL) levels were elevated from 4 weeks after treatment (WMD = 6.665, 95%CI = 3.906, 9.424, *P* < 0.001) to 24 weeks after treatment (WMD = 3.159,95% CI = 0.176, 6.142, *P* = 0.038). Triglyceride (TG) levels showed no significant change after the treatment.

**Conclusions:**

Hepatitis C patients who achieved SVR on DAAs showed the increase of lipid levels and the improvement of hepatic inflammation indicators AST and ALT. This may provide evidence-based medical evidence for the follow-up and monitoring of blood lipids and hyperlipidemia treatment.

**Registration:**

PROSPERO CRD42020180793.

**Supplementary Information:**

The online version contains supplementary material available at 10.1186/s12944-023-01957-2.

## Background

Hepatitis C virus (HCV) is a plus-strand RNA virus whose infection is mainly confined to liver cells and is an important cause of cirrhosis, liver cancer, and liver transplantation [1]. In 2019, the World Health Organization reported that about 58 million people worldwide are infected with chronic hepatitis C [2].

Studies have found that infection with HCV can affect lipid and lipoprotein metabolism levels in the body [3]. The typical presentation is enhanced lipid production and decreased lipoprotein secretion, which accelerates the process of atherosclerosis and liver steatosis [4, 5]. Moreover, clinical studies have found that the prevalence of liver steatosis in CHC patients is 40–86%, which is much higher than 20–50% of other chronic liver disease patients without hcv infection [6, 7]. Therefore treatment of HCV may be crucial in regulating the lipid metabolic disorders it causes. Sustained viral response (SVR) is defined as the disappearance of HCV RNA in plasma at 12 or 24 weeks after completion of treatment [8]. Before the advent of direct-acting antiviral drugs (DAAs), hepatitis C was mainly based on interferon (IFN), but its SVR rate was only about 50%, with serious side effects [9]. Recently developed DAAs are emerging as a new branch of standard HCV therapy that can significantly improve treatment outcomes [3]. Scott A McDonald et al. [10].

Patients with decompensated cirrhosis who received no interferon prior to the advent of DAA were compared and analysed with those who received no interferon DAA in the era of DAA. Patients with decompensated cirrhosis in the DAA era have a significantly lower risk of liver-related death.

In addition, Tanaka et al. [11]. found that DAA administration after hepatectomy could improve liver function in patients with HCC, which may prolong postoperative survival. Moreover, the study also found that DAA can adversely affect lipid profiles by eradicating HCV, which increases the risk of cardiovascular disease development. However, SVR can ultimately improve overall cardiovascular mortality by eliminating many other harmful effects of HCV [12].Therefore, it is particularly important to further understand the influence of DAAs treatment on the lipid profile of patients who achieved a sustained viral response to HCV.

Currently, there are many domestic and foreign studies on the relationship between DAAs treatment and lipid parameters in HCV patients. But the results are not identical. In 2018, Kawagishi N et al. [13] successfully eliminated HCV with interferon-free DAAs reducing low-density lipoprotein cholesterol (LDL-C) levels in patients with higher baseline values and in patients with hepatic steatosis and dyslipidemia in SVR24. Increased LDL-C levels are accompanied by increased sdLDL-C (Small and dense LDL-C) levels.

Kawagishi N et al. [13] successfully eliminated HCV with interferon-free DAAs in 2018, reducing low-density lipoprotein cholesterol (LDL-C) levels in SVR24 patients with higher baseline values and mid-hepatic steatosis and dyslipidemia. Elevated LDL-C levels are accompanied by increased levels of sdLDL-C (small, dense LDL-C).

However, Pedersen et al. [14] found that successful DAA treatment could increase LDL and High-density lipoprotein (HDL). In contrast, Triglyceride (TG) levels were reduced after treatment. This meta-analysis further explored the effect of DAA treatment on lipid levels from the perspective of a comprehensive assessment of the effect of persistent hepatitis C virus response on lipid parameters. In particular, the duration of lipid changes and the changes of lipid in patients with different genotypes or different SVR. So as to provide a reference for clinicians to individualized treatment.

## Materials and methods

This meta-analysis followed the PRISMA guidelines [15]. The search strategies and inclusion and exclusion criteria were registered with PROSPERO (PROSPEROCRD 42,020,180,793).

### Search strategies

The Medical Subject Heading terms and keywords used in the search process mainly included: “Hepatitis C,”“Hepacivirus,”“Sofosbuvir,”“DAA,”“Lipid Metabolism,”“Cholesterol,”“Triglyceride,”“Cholesterol, HDL,”“Cholesterol, LDL,” and “Apolipoproteins.”The databases searched included PubMed, Central, Embase, Web of Science. The search period was September 2023.

### Inclusion and exclusion criteria

The inclusion criteria:(1) The subjects of studies were patients with HCV; (2) studies on HCV patients who received DAA therapy; (3) availability of relevant lipid data before and after treatment; (4) studies in which persistent viral responses in HCV patients were clear and (5) prospective or retrospective studies. The exclusion criteria: (1) the study population was co-infected with HIV/HCV; (2) interferon was included in the treatment regimen; (3) text type: reviews, editorials, letters, case reports, personal newsletters, pre-prints and abstracts. Literature screening and data extraction were conducted independently by two researchers. First, primary literature retrieval was carried out, and then the literature retrieved at the primary level was screened according to pre-set criteria. When two researchers had different opinions, they discussed and settled together to reach a unified standard.

### Quality evaluation of literature evidence

The Newcastle Ottawa Scale (NOS) was used to assess study quality. We assessed the quality of the evidence for each relevant study. (The total score is 9 points. 7-9points: High-quality; 4–6 points Moderate-quality; 0–3 points Low-quality)

### Data extraction

Two independent researchers conducted the data extraction according to the formulated unified and standardized data tables. Relevant experts were invited to review these controversial issues. Data were extracted based on the following parameters: First author, Publication year, Country or region, Study type, Age, Percentage of males, DAAs protocol, Genotype, Sample size, SVR status, Baseline lipid parameters, and Lipid parameters at and after treatment (12 weeks, 24 weeks or one year). Mean ± standard deviation (SD) or median (interquartile spacing) was used to express age parameters and sex information was expressed as the percentage of males in each group.

### The primary outcome

(1) Changes in TG, total cholesterol (TC), LDL and HDL levels after DAAs treatment (at the end treatment, 4w after treatment, 12w after treatment, 24w after treatment, and one year after treatment) in patients who achieved sustained viral response compared with baseline; (2) Changes in lipids of patients with different SVR (SVR12 or SVR24) or different genotypes; (3) Changes of indicators of hepatic inflammation (AST and ALT ) before and after treatments; (4) In part of the study, changes of lipids in patients with cirrhosis and non-cirrhosis were analyzed.

### Statistical analysis

The software used in this study was Stata 12.0, and WMD values and the corresponding 95% confidence interval (CI) were used to measure and evaluate the association strength. Heterogeneity between studies was tested using the Q-test statistics and I^2^ values, and the I^2^ value was used to measure heterogeneity. Heterogeneity test I^2^ < 50% indicated no significant heterogeneity. Therefore, WMD was calculated using a fixed effects model. If heterogeneity was present, a random-effects model was selected. The Z statistic was used to test the combined WMD values. Differences with statistical significance was defined as P < 0.05. Egger’s linear regression method was used to evaluate publication bias. Sensitivity analysis was performed by eliminating individual studies individually.

## Results

### Search results

**Fig. 1:**
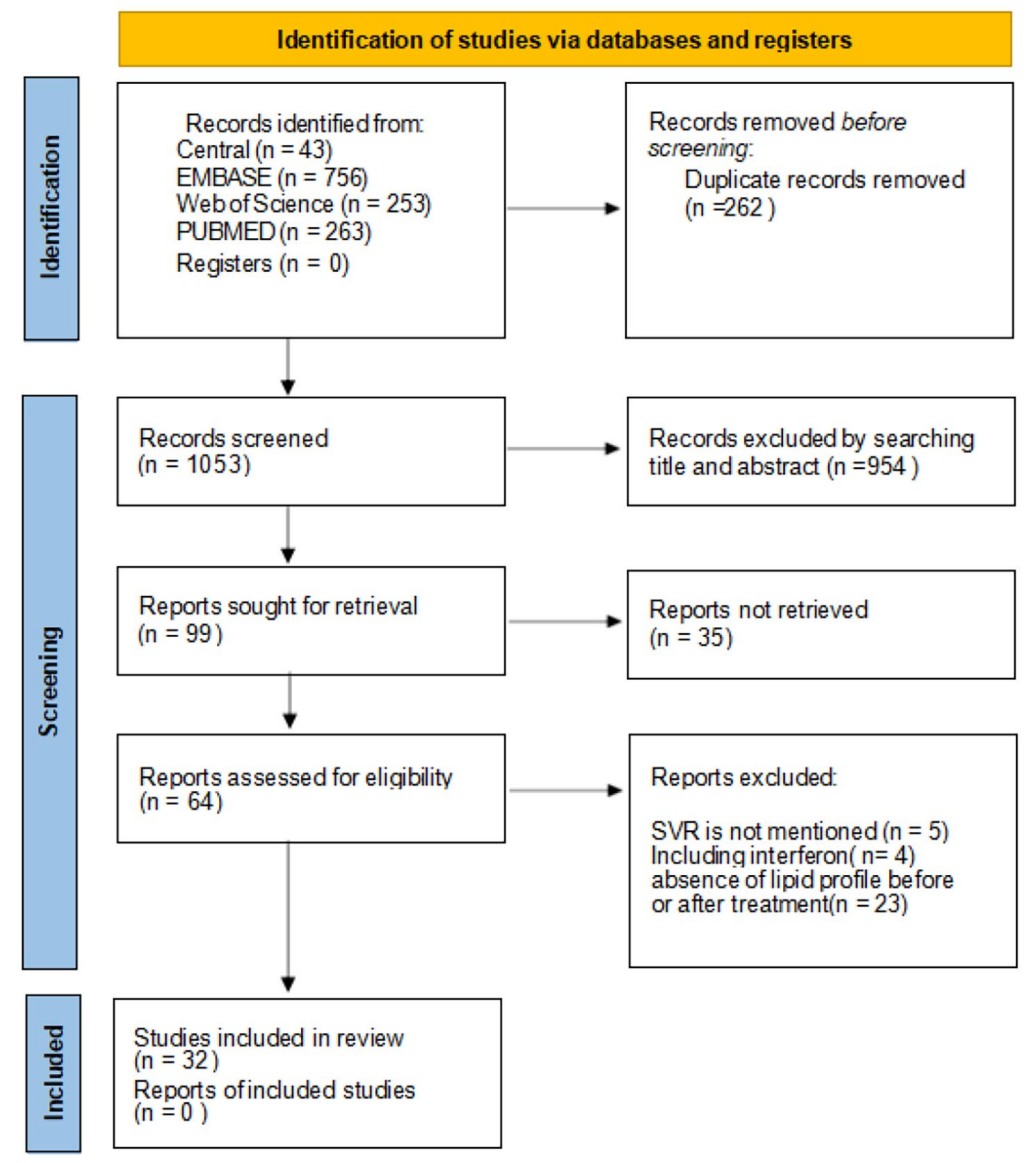
Flow diagram of study selection

A total of 1159 qualified studies were preliminarily retrieved according to the set retrieval formula, and 933 articles were retrieved that might be included in the study excluding 226 duplicate studies. After reading titles and abstracts, 848 irrelevant papers were excluded. 85 studies were excluded after reading the full text. Finally, 29 eligible papers were included **(**Fig. 1**).**

### Basic features of the included studies

Eleven studies were prospective studies, 13 were retrospective studies, one was retro-prospective study and seven types of studies were not mentioned in this meta-analysis; patients achieved SVR12 in twenty five studies and SVR24 in seven studies; one study reported changes in lipids in patients with significant and non-significant liver fibrosis, and three studies reported changes in lipids in patients with cirrhosis and non-cirrhosis. The research areas include Asia (India, Japan, Tai Wan), South America (Brazil), Europe (Italy, Spain, Germany), Africa (Egypt), North America (America, Canada) and Oceania (New Zealand). The basic characteristics are summarized in Table 1.

**Table 1:** Characteristics of Studies and patients

First author Year	Country/ Region	Study design	Antiviral regimens	Genotype	Weeks of treatment	SVR(W)	Number	Age (Years)	Males
Jain [16] 2018	India	prospective	SOF/DCV	G3	12	SVR12	47	38 ± 13	60%
Ichikawa [17] 2019	Japan	retrospective	DCV/ASV	G1b	24	SVR24	38	70.92 ± 11.02	36%
Ichikawa [18] 2019	Japan	retrospective	Multiple DAA regimens	Multiple genotypes	12/24	SVR12	48	70.1 ± 11.08	42%
Cheng [19] 2018	Tai wan	prospective	SOF/DCV	G2	12	SVR12	31	65.0 ± 13.2	28.10%
Cheng [20] 2019	Tai wan	prospective	Multiple DAA regimens	Multiple genotypes	12/24	SVR12	102	66.0 ± 10.7	33.30%
							AF 76	68.0 ± 9.3	32.90%
							NAF 26	60.3 ± 12.6	34.6%
Gilmar [21] 2018	Brazil	retrospective	Multiple DAA regimens	Multiple genotypes	12/24	SVR12	43	60.5 ± 9.5	27.90%
Inoue [22] 2018	Japan	NA	DCV/ASV	G1b	24	SVR24	69	68.3 ± 10.5	38.80%
			SOF/LDV	G1b	12	SVR24	84	64.4 ± 13.3	43.50%
			SOF/RBV	G2	12	SVR24	45	62.0 ± 15.3	32.60%
Gitto [23] 2018	Italy	NA	Multiple DAA regimens	Multiple genotypes	12/24	SVR24	93	64 ± 12	60.20%
El Sagheer [24] 2018	Egypt	NA	SIM/SOF	G4	12	SVR12	79	47 ± 12	58.80%
Chida [25] 2018	America	retrospective	DCV/ASV	G1b	24	SVR12	67	71 ± 9	40.00%
Andrade [26] 2018	Brazil	retro- prospective	Multiple DAA regimens	Multiple genotypes	12	SVR12	95	56 ± 9	70%
Juanbeltz [27] 2017	Spain	retrospective	Multiple DAA regimens	Multiple genotypes	12/16/24	SVR12	212	53.6 ± 9.3	71.80%
Endo [28] 2017	Japan	NA	DCV/ASV	G1b	24	SVR12	121	68.4 ± 11.8	48.80%
			SOF/LDV	G1b	24	SVR12	132	66.7 ± 13.1	37.10%
Pedersen [14] 2016	America	prospective	SOF/RBV	G2	12/24	SVR12	58	55.5 ± 12.1	61.20%
							C 33		
							NC 25		
			SOF/RBV	G3	12/24	SVR12	31	54.6 ± 10.8	64.50%
							C 14		
							NC 17		
Shimizu [29] 2018	Japan	NA	Multiple DAA regimens	G1/G2	12/24	SVR12	70	66 (59–73)	41.40%
Beig [30] 2018	New Zealand	retrospective	Multiple DAA regimens	NA	NA	SVR12	35	NA	NA
Sun [31] 2017	Tai wan	NA	GZR/EBV or SOF/LDV	G1	12	SVR12	22	60 (39 ~ 83)	50%
Doyle [32] 2019	Canada	NA	PrOD	G1a/G1b	12	SVR12	23	54 ± 11.6	71%
Muñoz.H [33] 2020	NA	prospective	Multiple DAA regimens	Multiple genotypes	12	SVR12	109	53.6 ± 10.8	69.70%
Sangineto [34] 2021	Italy	retrospective	Multiple DAA regimens	NA	NA	SVR24	95	67.1 ± 0.8	50.60%
Inomata [35] 2022	Japan	retrospective	SOF/LDV	G1b	12	SVR12	22	60.5 (55–69)	50%
Graf [5] 2020	Germany	retrospective	Multiple DAA regimens	Multiple genotypes	12	SVR24	45	51.7 ± 14.1	47.80%
Chen [36] 2020	Tai wan	prospective	Multiple DAA regimens	Multiple genotypes	12/24	SVR12	102	65.9 ± 9.9	32.40%
Iossa [37] 2021	Italy	retrospective	Multiple DAA regimens	Multiple genotypes	12	SVR24	47	66 (62–71)	42.90%
							C 31		
							NC 18		
Eletreby [38] 2021	Egypt	prospective	SOF/DAC ± RBV	Multiple genotypes	12/24	SVR12	264	51.73 ± 10.24	89.20%
Nevola [39] 2020	Italy	prospective	Multiple DAA regimens	Multiple genotypes	8–24	SVR24	243	68 (62–74)	46.90%
Joshita [40] 2021	Japan	retrospective	Multiple DAA regimens	Multiple genotypes	8/12/24	SVR12	231	70 (63–76)	42.00%
Abdoa [41] 2020	Egypt	retrospective	SOF/DCV	NA	12/24	SVR12	98	51.54 ± 6.91	44.90%
							C 32		
							NC 66		
Hino [42] 2021	NA	retrospective	Multiple DAA regimens	Multiple genotypes	12	SVR12	67	70.0 (62–77)	40.30%
Anca [43] 2023	Italy	retrospective	Multiple DAA regimens	NA	12	SVR12	132	61.17 ± 9.11	35.6%
Ahmed [44] 2023	Egypt.	retrospective	Multiple DAA regimens	NA	12	SVR12	100	50.99 ± 8.75	100%
Diego [45] 2023	Spain	retrospective	Multiple DAA regimens	Multiple genotypes	8/12	SVR12	83	55 (49–63)	49.4%

NA,not available; AF,Advanced fibrosis; NAF,Non-advanced fibrosis;C,Cirrhotics; NC,Non-cirrhotics;

SOF: Sofosbuvir; DCV: Daclatasvir;ASV: Asunaprevir;LDV: Ledipasvir;

LDV: Ledipasvir;RBV: Ribavirin;SIM: Simeprevir;GZR: Grazoprevir;

EBV: Elbasvir; PrOD: paritaprevir/ritonavir/ombitasvir/dasabuvir;DAC: Daclatasvir

### Quality evaluation of literature evidence

NOS scores were performed on the 32 included studies, and the results showed that the scores of all studies were no less than six, indicating that the 32 studies were of.

medium and high quality. **(Supplementary Table** 1).

### Change in TC after antiviral therapy

**Fig. 2:**
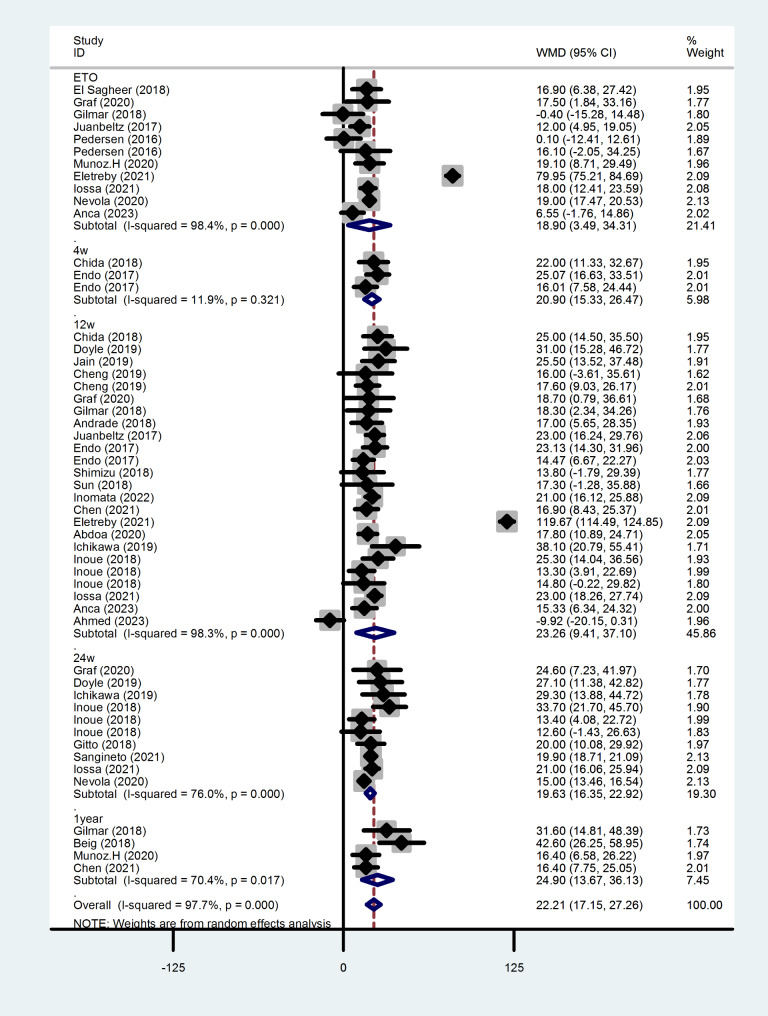
Forest plot of serum TC changes after treatment at each time point

Figure 2 shows the changes in total cholesterol levels in patients who achieved a sustained viral response after DAAs treatment. Results show that the TC level increased at the end of treatment (WMD = 18.905, 95%CI = 3.495, 34.314,*P* = 0.016), 4 weeks after completion of treatment (WMD = 20.901, 95%CI = 15.335, 26.468, *P* < 0.001), 12 weeks (WMD = 23.255, 95%CI = 9.414, 37.096, *P* = 0.001), 24 weeks (WMD = 19.635, 95% CI = 16.353, 22.917, *P* < 0.001) and one year (WMD = 24.900, 95% CI = 13.669, 36.131, *P* < 0.001) after treatment compared with that before the treatment. Because of the high heterogeneity of the study, sensitivity analyses were performed and the results were found to be stable. And there was no significant publication bias, so a random-effects model was appropriate for statistical analysis.

### Change in LDL after antiviral therapy

**Fig. 3:**
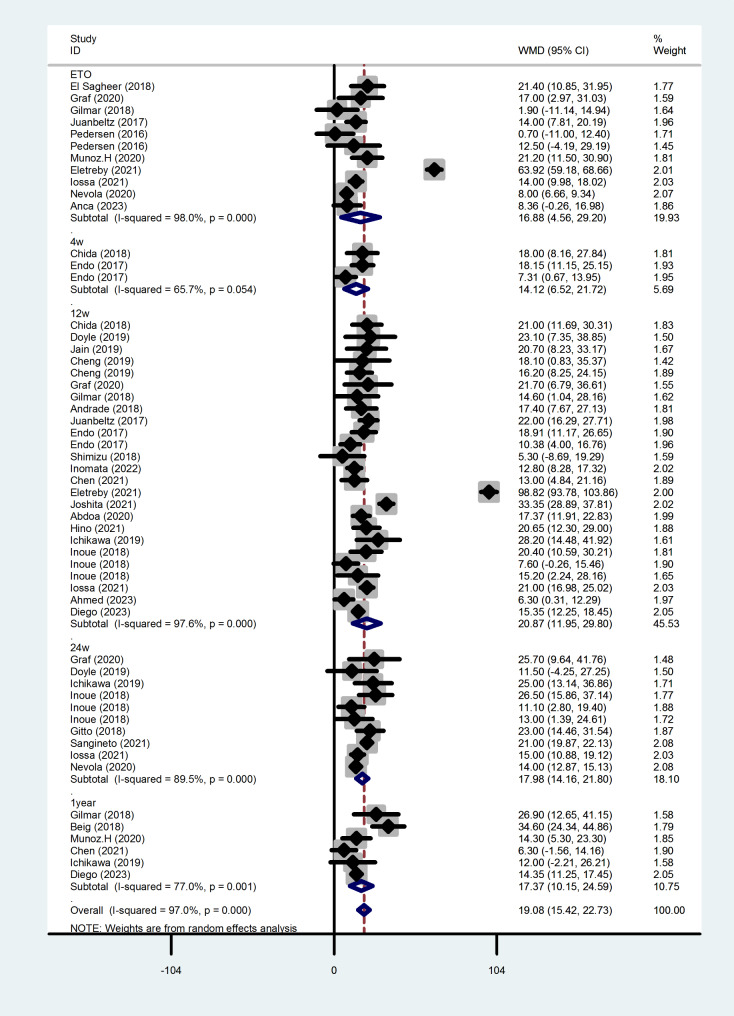
Forest plot of serum LDL changes after treatment at each time point

Figure 3 shows the changes in LDL levels in HCV patients after DAAs treatment ended. Compared with before of treatment, serum LDL levels increased from the end of treatment (WMD = 16.88, 95%CI = 4.564, 29.195,*P* = 0.007) to 1 year after treatment (WMD = 17.372, 95%CI = 10.152, 24.592, *P* < 0.001). Heterogeneity was the same as that for TC, and there was no significant publication bias.

### Change in HDL after antiviral therapy

**Fig. 4:**
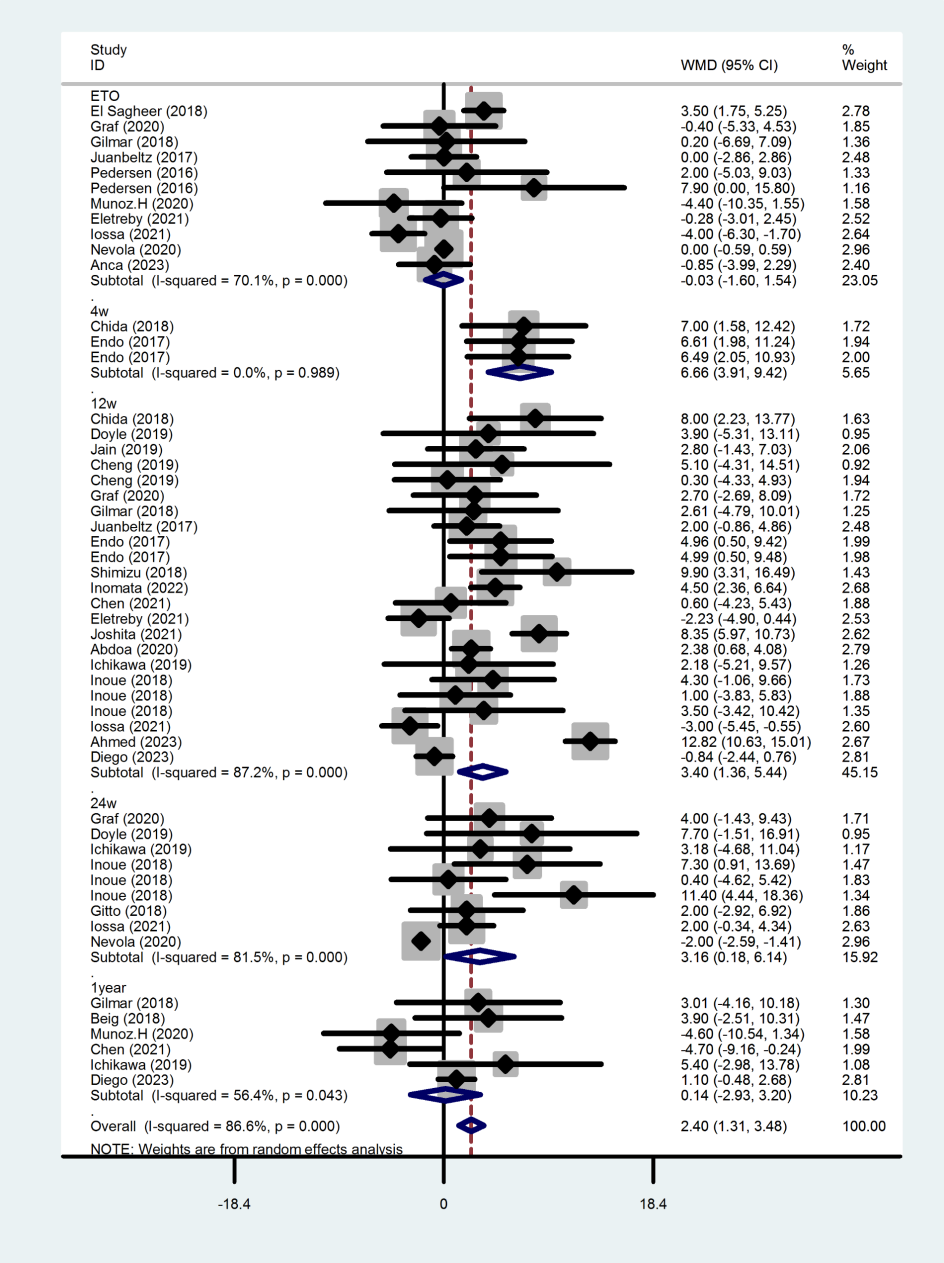
Forest plot of serum HDL changes after treatment at each time point

As Fig. 4 shows, the levels of HDL from 4 weeks after treatment (WMD = 6.665, 95%CI = 3.906–9.424, *P* < 0.001) to 24 weeks after treatment (WMD = 3.159, 95%CI = 0.176–6.142, *P* = 0.038) were elevated compared with that before the treatment. However, at the end of treatment (WMD=-0.030, 95%CI =-1.595-1.536, ***P*** = 0.970) and one year after the end of treatment (WMD = 0.136, 95%CI = -2.929-3.200, *P* = 0.931), the changes of HDL levels were not significant.

ication bias, so a trim and fill analysis was carried out. Before the trim-and-fill test, the heterogeneity test was Q = 381.642, *P* < 0.001, and the random effect model was adopted. The combined effect size result was 2.397, 95% CI = 1.308–3.485, and the heterogeneity test after the trim-and-fill Q = 718.837, *P* < 0.001.The combined effect size was 0.844 (with 95% CI = 0.270–2.644). The results were not reversed before and after trim-and-fill analysis, indicating that the results were relatively robust.

#### Change in TG after antiviral therapy

**Fig. 5:**
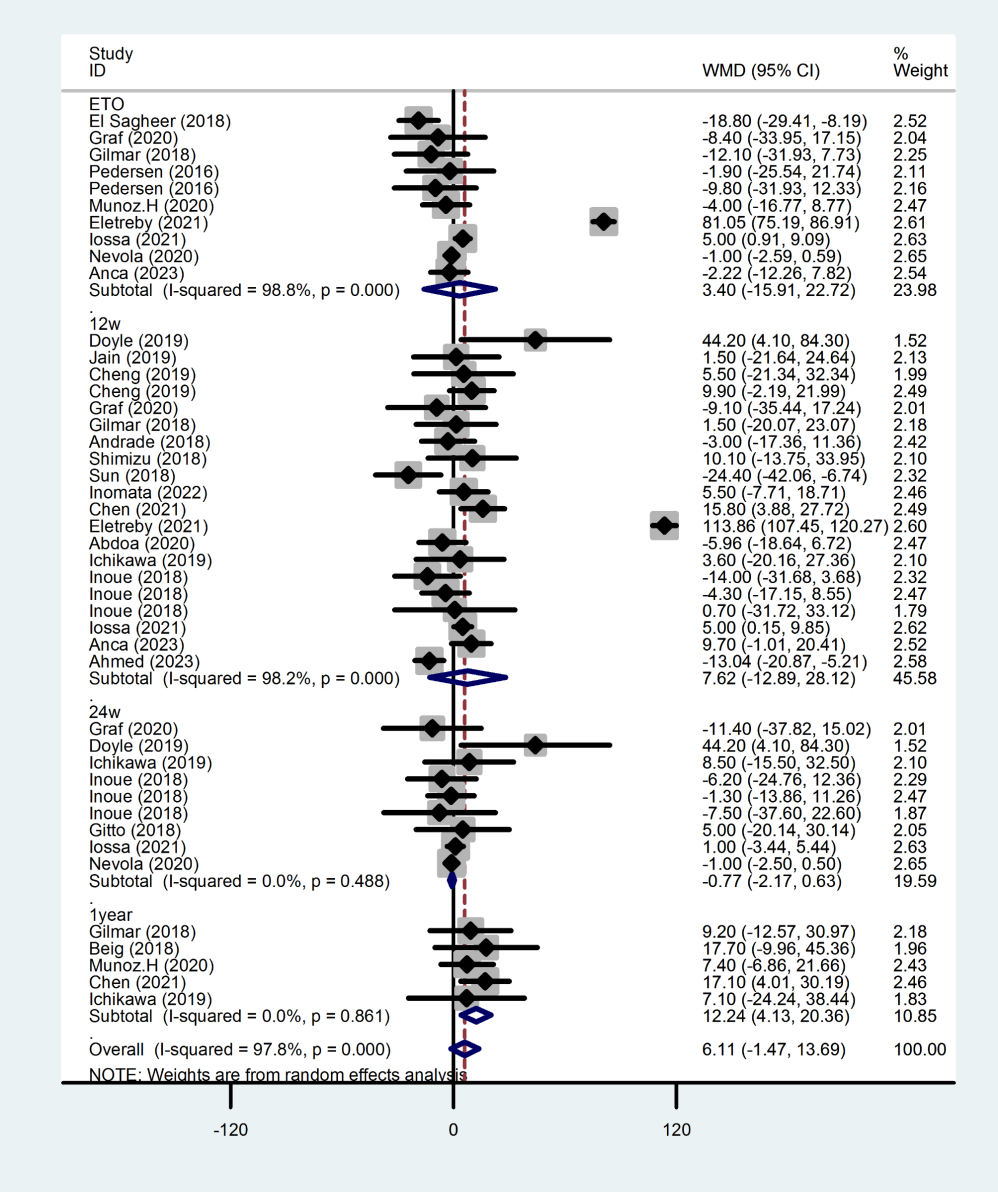
Forest plot of serum TG changes after treatment at each time point

Figure 5 shows the changes of TG in patients before and after treatment, and there was no statistically significant at the end of treatment (WMD = 3.403, 95%CI =-15.915-22.721, *P* = 0.730), 12 weeks after completion of treatment (WMD = 7.616, 95%CI =-12.893, 28.1248, *P* = 0.467), 24 weeks after completion of treatment (WMD= -0.772,95% CI=-2.170,0.626, *P* = 0.279).

### Subgroup analysis was performed according to different SVR

Supplementary Figs. 1 and 2 show the changes in TC and LDL levels in patients with different SVR. The results showed that in patients who achieved SVR12 and SVR24,the serum TC levels (SVR12: WMD = 22.743, 95%CI = 11.064, 34.423, *P* < 0.001; SVR24: WMD = 19.401, 95% CI = 17.335,21.468, *P* < 0.001;) and LDL levels (SVR12: WMD = 19.612, 95%CI = 12.253, 26.971, *P* < 0.001; SVR24: WMD = 17.017, 95% CI = 13.735,20.298, *P* < 0.001;) increased after treatment. There was no publication bias.

### Subgroup analysis was performed according to different genotypes

Supplementary Fig. 3 shows the changes in TC and LDL levels in the patients with different genotypes. The results showed that serum TC and LDL levels of patients with the G1b, G1, G2, and G3 genotypes increased after treatment, with no publication bias. (**Supplementary Table** 2).

### Change of AST and ALT

Seven studies reported changes in AST levels before and after treatment. AST levels decreased after treatment compared to before treatment (WMD=-27.339, 95%CI = -35.294, -19.385, *P* < 0.001), as shown in Supplementary Fig. 4. Ten studies reported changes in ALT before and after treatment, and ALT levels also decreased after treatment (WMD=-40.820, 95%CI = -49.872, -31.767, *P* < 0.001), as shown in Supplementary Fig. 5. There was a publication bias in both cases. After two iterations, there were no missing articles, and the research results were relatively reliable.

### Change in lipids in patients with Cirrhosis and non-cirrhosis

Three studies reported changes in lipid levels in patients with cirrhosis and non-cirrhosis. Pooled analysis showed that TC in cirrhosis and non-cirrhosis patients (cirrhosis: WMD = 13.824, 95% CI = 7.310, 20.337, *P* < 0.001; non-cirrhosis: WMD = 17.139, 95%CI = 10.601,23.676, *P* < 0.001) and LDL (cirrhosis: WMD = 8.498, 95%CI = 3.474, 13.522, *P* = 0.001;non-cirrhosis: WMD = 17.702, 95%CI = 12.349, 23.67623.054, *P* < 0.001) after treatment was increased compared with that before treatment, as shown in Supplementary Figs. 6 and 7. TG and HDL levels were not statistically significant in either population, and there was no significant heterogeneity or publication bias.

## Discussion

This study aims to show lipid changes in HCV patients who achieve SVR after DAA treatment and persistent changes in blood lipid levels within 1 year after DAA treatment.

Currently, the mechanism of the interaction and influence between HCV and blood lipids remains unclear. Previous studies have shown that inhibition of cholesteryl ester and triglyceride synthesis can inhibit viral infection by inhibiting the assembly process of hepatitis C virus [46].On the other hand, hepatitis C virus itself is a high-fat lipovirus particle, very similar to very low-density lipoprotein(VLDL), which can alter liver lipoprotein-related functions in a variety of ways, including by impairing the VLDL release pathway. Therefore, it is related to the accumulation of liver lipids and the pathogenesis of dyslipidemia [47]. Additionally, it can enhance replication by regulating host cell lipid metabolism [48]. In HCV patients who have achieved SVR, HCV RNA cannot be detected in the plasma, which may reduce lipid metabolism in the host and affect the patient’s lipid levels. Currently, there are many treatments for HCV infection, but compared with traditional peginterferon-based treatment regimens, recent DAAs have increased the persistent viral response rate in HCV patients [49]. Furthermore, studies also have found that DAA treatment in HCV patients can lead to good cardiovascular outcomes while reducing the potential for insulin resistance and diabetes development [50–52] .

There have been many studies on HCV treatment and lipid changes, but the results have not been the same. Stefan et al. [53] found that suppression and elimination of HCV by DAAs without interferon had no effect on TG but increased TC levels; however, interferon-based therapy increased TG and TC during treatment and led to elevated TC when a sustained virological response was achieved. DAAs therapy for hepatitis C is closely related to lipid changes in patients [31].

In 2021, RosannaVillani et al. [54]. conducted a meta-analysis in which they examined changes in blood lipid levels during DAA treatment and at 12 and 24 weeks after the end of treatment. The results showed that the patient’s TC, LDL, and HDL levels increased, which persisted for 24 weeks after the end of treatment. The differences between this study and that of RosannaVillani et al. are as follows:1. Study population: the study population of Rosanna Villani et al. included patients treated with DAA, while this study targeted patients who achieved SVR after DAA treatment, which can exclude the effect of SVR on patients’ blood lipids; 2. Observation time: R et al. analyzed the changes in blood lipids of patients from the treatment period to 24 weeks after treatment; this study was extended to 1 year after the end of treatment to observe further the long-term effects of DAA on patients’ blood lipids. 3. Subgroup analysis: Further subgroup analysis was performed for different sustained viral response times and different genotypes in this study. 4. The inclusion of more studies in this meta-analysis made the results more robust.

Finally, the meta-analysis included 32 articles showing a correlation between persistent hepatitis C virus response and lipid changes. Analytical data showed that serum TC and LDL levels in HCV patients were significantly elevated from the end of treatment to one year after the end of treatment. At the same time, within 4 to 24 weeks after the end of DAA treatment, the patient’s serum HDL level also increased significantly, but the TG level did not change significantly. In addition, AST and ALT levels also improved after treatment.

It can be seen that after hepatitis C patients achieve SVR by DAA treatment, the main changes in blood lipids are persistent increases in TC, LDL and HDL, while TG has no significant change. This may provide a reference for the treatment of lipid-lowering in HCV patients and the long-term detection of lipids.

Hepatitis C has a variety of genotypes, in this study, by analyzing the changes of lipids in patients with different genotypes who obtained SVR, it can be found that the levels of serum TC and LDL in patients with G1b, G1, G2 and G3 genotypes were all increased after treatment. Similarly, Jain et al. [55] demonstrated a significant increase in TC and LDL with SVR in HCV genotype 3 patients. And Doyle et al. [56] also found a significant increase in TC and LDL levels after achieving SVR in a study of genotype 1 patients. Antiviral therapy affects lipid metabolism [57], and the differences in the effects of different genotypes on blood lipids in patients with SVR seem to be inconclusive at present, which may require further research in the future.

In addition, a sensitivity analysis was conducted by excluding single studies. The sensitivity analysis did not affect the combined effect size by excluding single studies, suggesting that the results of the meta-analysis were robust.

### Strengths and limitations

This study comprehensively analyzed the lipid changes in patients who achieved SVR after DAA treatment. The changes of lipid in patients with different genotypes and different SVR were also compared.

In addition, this study had some limitations. First, only English databases were selected for literature retrieval; Therefore, the scope of the selected literature was not wide enough, and the number of included studies was small. Further high-quality studies with larger samples size are required. Second, in some studies of this study showed significant heterogeneity. Although sensitivity analysis was conducted and a random effects model was finally adopted for analysis, the stability of the meta-analysis results may be affected to a certain extent. Third, there was a publication bias in part of the analysis process, which was identified and processed. Fourth, the age of the research population included in the literature is between 50 and 70 years, and more studies on other age groups are needed.

## Conclusion

In summary, this meta-analysis suggests that the sustained viral response induced by DAAs treatment in HCV patients is significantly associated with increased serum TC, LDL, and HDL levels and improvements in AST and ALT levels after treatment.

There were similar changes in serum TC and LDL levels in patients with and without cirrhosis. which provided a reference value for long-term lipid-lowering therapy in patients. Future research may focus on these changes and the choice of lipid-lowering therapies to reduce the incidence of fatty liver and cardiovascular disease.

**Declarations**.

### Electronic supplementary material

Below is the link to the electronic supplementary material.

Supplementary Material 1

Supplementary Material 2

Supplementary Material 3

Supplementary Material 4

Supplementary Material 5

## Data Availability

The datasets used and/or analysed during the current study are available from the. corresponding author on reasonable request.

## Abbreviations

DAAs: Direct-acting Antiviral drugs
HCV: Hepatitis C virus
SVR: Sustained Viral Response
TC: Total cholesterol
LDL: Low-density lipoprotein
HDL: High-density lipoprotein
TG: Triglyceride
IFN: interferon
NOS: Newcastle Ottawa Scale
NA: Not available
AF: Advanced fibrosis
NAF: Non-advanced fibrosis
C: Cirrhotics
NC: Non-cirrhotics
SOF: Sofosbuvir
DCV: Daclatasvir
ASV: Asunaprevir
LDV: Ledipasvir
LDV: Ledipasvir
RBV: Ribavirin
SIM: Simeprevir
GZR: Grazoprevir
EBV: Elbasvir
PrOD: Paritaprevir/ritonavir/ombitasvir/dasabuvir
DAC: Daclatasvir

## References

[1] Popescu CI, Dubuisson J (2009). Role of lipid metabolism in Hepatitis C virus assembly and entry[J]. Biol Cell.

[2] Hepatitis C. WHO https://www.who.int/news-room/fact-sheets/detail/hepatitis-c.

[3] Felmlee DJ, Hafirassou ML, Lefevre M (2013). Hepatitis C virus, cholesterol and lipoproteins–impact for the viral life cycle and pathogenesis of Liver disease[J]. Viruses.

[4] Lanini S, Scognamiglio P, Pisapia R (2019). Recovery of metabolic impairment in patients who cleared chronic Hepatitis C Infection after direct-acting antiviral therapy[J]. Int J Antimicrob Agents.

[5] Graf C, Welzel T, Bogdanou D et al. Hepatitis C clearance by direct-acting antivirals impacts glucose and lipid Homeostasis[J]. J Clin Med, 2020,9(9). 10.3390/jcm9092702 PMC7564474 32825571

[6] Lonardo A, Loria P, Adinolfi LE (2006). Hepatitis C and steatosis: a reappraisal [J]. J Viral Hepat.

[7] Serfaty L, Andreani T, Giral P (2001). Hepatitis C virus induced hypobetalipoproteinemia: a possible mechanism for steatosis in chronic Hepatitis C[J]. J Hepatol.

[8] Kohli A, Shaffer A, Sherman A (2014). Treatment of Hepatitis C: a systematic review[J]. JAMA.

[9] Pawlotsky JM (2014). New Hepatitis C therapies: the toolbox, strategies, and challenges[J]. Gastroenterology.

[10] McDonald SA, Barclay ST, Innes HA (2021). Uptake of interferon-free DAA therapy among HCV-infected decompensated Cirrhosis patients and evidence for decreased mortality[J]. J Viral Hepat.

[11] Tanaka S, Shinkawa H, Tamori A (2021). Postoperative direct-acting antiviral treatment after liver resection in patients with Hepatitis C virus-related hepatocellular carcinoma[J]. Hepatol Re.

[12] Roguljic H, Nincevic V, Bojanic K (2021). Impact of DAA Treatment on Cardiovascular Disease Risk in Chronic HCV Infection: an Update[J]. Front Pharmacol.

[13] Kawagishi N, Suda G, Nakamura A (2018). Liver steatosis and dyslipidemia after HCV eradication by direct acting antiviral agents are synergistic risks of atherosclerosis[J]. PLoS ONE.

[14] Pedersen MR, Patel A, Backstedt D (2016). Genotype specific peripheral lipid profile changes with Hepatitis C therapy[J]. World J Gastroenterol.

[15] Page MJ, McKenzie JE, Bossuyt PM (2021). The PRISMA 2020 statement: an updated guideline for reporting systematic reviews. Rev Esp Cardiol (Engl Ed).

[16] Jain A, Kalra BS, Srivastava S (2019). Effect of sofosbuvir and daclatasvir on lipid profile, glycemic control and quality of life index in chronic Hepatitis C, genotype 3 patients[J]. Indian J Gastroenterol.

[17] Ichikawa T, Miyaaki H, Miuma S (2019). Changes in serum LDL, PCSK9 and microRNA-122 in patients with chronic HCV Infection receiving Daclatasvir/Asunaprevir[J]. Biomed Rep.

[18] Ichikawa T, Miyaaki H, Miuma S (2019). Carotid intima-media thickness and small dense low-density lipoprotein cholesterol increase after one year of treatment with direct-acting antivirals in patients with Hepatitis C Virus Infection[J]. Intern Med.

[19] Cheng PN, Chiu YC, Chien SC (2019). Real-world effectiveness and safety of sofosbuvir plus daclatasvir with or without Ribavirin for genotype 2 chronic Hepatitis C in Taiwan[J]. J Formos Med Assoc.

[20] Cheng PN, Chen JY, Chiu YC (2019). Augmenting central arterial stiffness following eradication of HCV by direct acting antivirals in advanced fibrosis patients[J]. Sci Rep.

[21] Lacerda GS, Medeiros T, Rosario N (2018). Exploring lipid and apolipoprotein levels in chronic Hepatitis C patients according to their response to antiviral treatment[J]. Clin Biochem.

[22] Inoue T, Goto T, Iio E (2018). Changes in serum lipid profiles caused by three regimens of interferon-free direct-acting antivirals for patients infected with Hepatitis C virus[J]. Hepatol Res.

[23] Gitto S, Cicero A, Loggi E (2018). Worsening of serum lipid Profile after Direct Acting Antiviral Treatment[J]. Ann Hepatol.

[24] El SG, Soliman E, Ahmad A (2018). Study of changes in lipid profile and insulin resistance in Egyptian patients with chronic Hepatitis C genotype 4 in the era of DAAs[J]. Libyan J Med.

[25] Chida T, Kawata K, Ohta K (2018). Rapid Changes in serum lipid profiles during combination therapy with Daclatasvir and asunaprevir in patients infected with Hepatitis C Virus genotype 1b[J]. Gut Liver.

[26] Andrade VG, Yamashiro F, Oliveira CV (2018). INCREASE OF LIPIDS DURING HCV TREATMENT: VIRUS ACTION OR MEDICATION?[J]. Arq Gastroenterol.

[27] Juanbeltz R, Goni ES, Uriz-Otano JI (2017). Safety of oral direct acting antiviral regimens for chronic Hepatitis C in real life conditions[J]. Postgrad Med.

[28] Endo D, Satoh K, Shimada N (2017). Impact of interferon-free antivirus therapy on lipid profiles in patients with chronic Hepatitis C genotype 1b[J]. World J Gastroenterol.

[29] Shimizu K, Soroida Y, Sato M (2018). Eradication of Hepatitis C virus is associated with the attenuation of steatosis as evaluated using a controlled attenuation parameter[J]. Sci Rep.

[30] Beig J, Orr D, Harrison B (2018). Hepatitis C Virus Eradication with New Interferon-Free Treatment improves metabolic Profile in Hepatitis C virus-related liver transplant Recipients[J]. Liver Transpl.

[31] Sun HY, Cheng PN, Tseng CY (2018). Favouring modulation of circulating lipoproteins and lipid loading capacity by direct antiviral agents grazoprevir/elbasvir or ledipasvir/sofosbuvir treatment against chronic HCV infection[J]. Gut.

[32] Yang Y, Wu FP, Wang WJ (2019). Real life efficacy and safety of direct-acting antiviral therapy for treatment of patients infected with Hepatitis C virus genotypes 1, 2 and 3 in northwest China[J]. World J Gastroenterol.

[33] Munoz-Hernandez R, Ampuero J, Millan R (2020). Hepatitis C Virus Clearance by Direct-Acting antivirals agents improves endothelial dysfunction and subclinical Atherosclerosis: HEPCAR Study[J]. Clin Transl Gastroenterol.

[34] Sangineto M, Luglio CV, Mastrofilippo T (2021). Analysis of hepatic stiffness after viral eradication in a population with chronic Hepatitis C treated with DAAs[J]. Med Clin (Barc).

[35] Inomata S, Morihara D, Anan A (2022). Male-specific association between Iron and lipid metabolism changes and Erythroferrone after Hepatitis C Virus Eradication[J]. Intern Med.

[36] Chen JY, Cheng PN, Chiu YC (2021). Persistent augmentation of central arterial stiffness following viral clearance by direct-acting antivirals in chronic Hepatitis C[J]. J Viral Hepat.

[37] Iossa D, Vitrone M, Gagliardi M (2021). Anthropometric parameters and liver histology influence lipid metabolic changes in HCV chronic hepatitis on direct-acting antiviral treatment[J]. Ann Transl Med.

[38] Eletreby R, Anees M, Naguib M (2021). The interrelation between lipid profile in chronic HCV patients and their response to antiviral agents[J]. Expert Rev Gastroenterol Hepatol.

[39] Nevola R, Rinaldi L, Zeni L (2020). Metabolic and renal changes in patients with chronic Hepatitis C Infection after Hepatitis C virus clearance by direct-acting antivirals[J]. JGH Open.

[40] Joshita S, Yamashita Y, Okamoto T (2021). Quantitative and qualitative lipid improvement with chronic Hepatitis C virus eradication using direct-acting antivirals[J]. Hepatol Res.

[41] Abdo M, Rabiee A, Abdellatif Z (2021). Impact of sustained virological response on metabolic disorders in diabetic chronic Hepatitis C virus patients after treatment with generic sofosbuvir and daclatasvir[J]. Eur J Gastroenterol Hepatol.

[42] Hino N, Sasaki R, Takahashi Y (2021). Treatment of Hepatitis C Virus Infection with direct-acting Antiviral agents elevates the serum small-dense low-density lipoprotein cholesterol Level[J]. Intern Med.

[43] Anca T, Tudor C, Robert N (2023). Changes in components of metabolic syndrome after antiviral eradication in Hepatitis C Virus Infection[J]. Life.

[44] Ahmed El, Tarek Y, Wesam I (2023). The efect of diferent direct antivirals on hepatic steatosis in nondiabetic and naïve hepatitis Cinfected Egyptian patients [J]. Egypt J Intern Med.

[45] Diego C, Silvia E, Ana M (2023). Triglyceride-rich lipoproteins and insulin resistance in patients with chronic Hepatitis C receiving direct-acting antivirals [J]. Atherosclerosis.

[46] Miyanari Y, Atsuzawa K, Usuda N (2007). The lipid droplet is an important organelle for Hepatitis C virus production[J]. Nat Cell Biol.

[47] Schaefer EA, Chung RT (2013). HCV and host lipids: an intimate connection[J]. Semin Liver Dis.

[48] Syed GH, Amako Y, Siddiqui A (2010). Hepatitis C virus hijacks host lipid metabolism[J]. Trends Endocrinol Metab.

[49] Jimenez GR, Albacete RA, Monje AP (2014). [New Drugs in the treatment of chronic Hepatitis C][J]. Farm Hosp.

[50] Butt AA, Yan P, Shuaib A (2019). Direct-acting antiviral therapy for HCV Infection is Associated with a reduced risk of Cardiovascular Disease Events[J]. Gastroenterology.

[51] Adinolfi LE, Nevola R, Guerrera B (2018). Hepatitis C virus clearance by direct-acting antiviral treatments and impact on insulin resistance in chronic Hepatitis C patients[J]. J Gastroenterol Hepatol.

[52] Roccaro GA, Mitrani R, Hwang WT (2018). Sustained virological response is Associated with a decreased risk of Posttransplant Diabetes Mellitus in Liver Transplant recipients with Hepatitis C-Related Liver Disease[J]. Liver Transpl.

[53] Mauss S, Berger F, Wehmeyer MH, et al. Effect of antiviral therapy for HCV on lipid levels[J]. Antivir Ther. 2017;21(1):81–88. 10.3851/IMP3094 27685337

[54] Villani R, Di Cosimo F, Romano AD (2021). Serum lipid profile in HCV patients treated with direct-acting antivirals: a systematic review and meta-analysis[J]. Sci Rep.

[55] Jain A, Kalra BS, Srivastava S, Chawla S (2019). Effect of sofosbuvir and daclatasvir on lipid profile, glycemic control and quality of life index in chronic Hepatitis C, genotype 3 patients[J]. Gastroenterol.

[56] Doyle MA, Galanakis C, Mulvihill E (2019). Hepatitis C direct acting antivirals and Ribavirin modify lipid but not glucose parameters. Cells.

[57] Meissner EG, Lee YJ, Osinusi A (2015). Effect of Sofosbuvir and Ribavirin treatment on peripheral and hepatic lipid metabolism in chronic Hepatitis C virus, genotype 1-infected patients. Hepatology.

