# Supplementary material for: Effects of sustained viral response on lipid in Hepatitis C: a systematic review and meta-analysis
Source: Lipids Health Dis. 2024 Mar 9;23:74. doi: 10.1186/s12944-023-01957-2 (PMC10924993; doi:10.1186/s12944-023-01957-2)
Supplement: Supplementary file 1 — Supplementary Material 1 [file 12944_2023_1957_MOESM1_ESM.docx]

Supplementary Figure 1 Changes of serum TC in patients

with different SVR after treatment. Forest plots with weighted

mean difference (WMD) and 95% confidence interval (CI).

Supplementary Figure 2 Changes of serum LDL in patients

with different SVR after treatment. Forest plots with

weighted mean difference (WMD) and 95% confidence interval (CI).


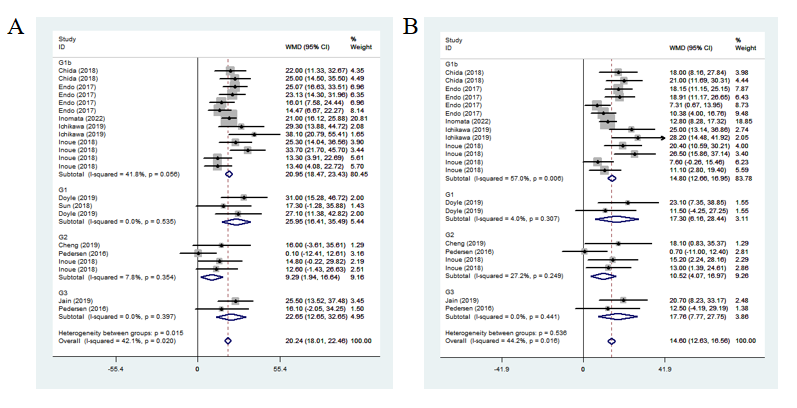


Supplementary Figure 3 Changes of serum TC and LDL in patients with different

genotypes after treatment. Forest plots with weighted mean difference (WMD) and 95% confidence interval (CI). A, The changes of TC; B, The changes of LDL.

Supplementary Figure 4 changes in serum AST after treatment,

forest plots of weighted mean difference (WMD) and 95% confidence interval (CI)

Supplementary Figure 5 changes in serum ALT after treatment,

forest plots of weighted mean difference (WMD) and

95% confidence interval (CI)


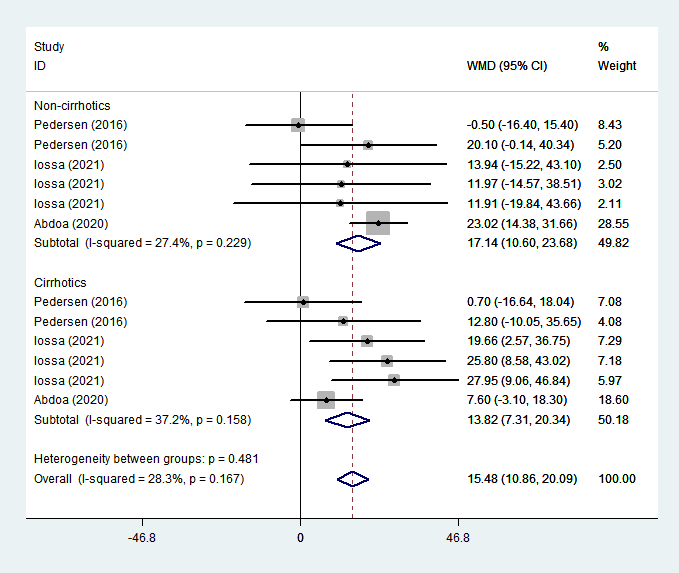


Supplementary Figure 6 Changes of serumTC in Cirrhosis and

Non-cirrhosis patientsafter treatment. Forest plots with weighted

mean difference (WMD)and 95% confidence interval(CI).


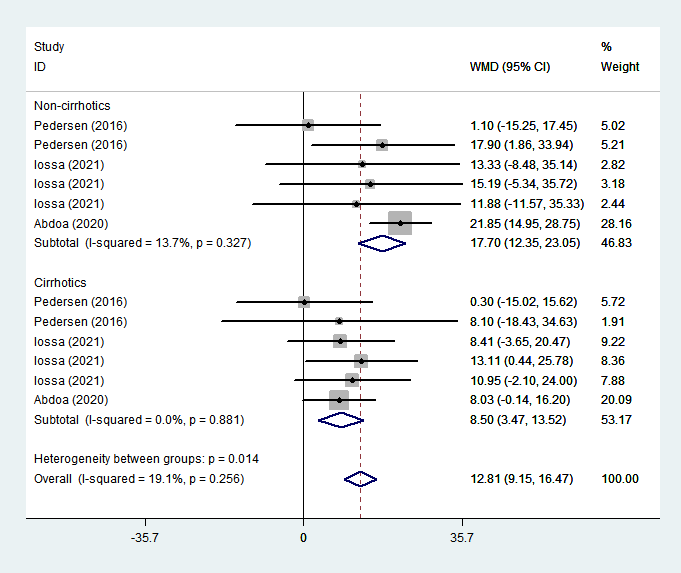


Supplementary Figure 7 Changes of serum LDL in Cirrhosis and

Non-cirrhosis patientsafter treatment. Forest plots with weighted

mean difference (WMD) and95% confidence interval(CI).

Supplementary Table 1. The quality evaluation of included studies

| Study | Selection | Comparability | Outcome | Overall quality |
| --- | --- | --- | --- | --- |
| Jain. 2019 | ☆☆☆ |  | ☆☆☆ | 6 |
| Ichikawa. 2019 | ☆☆☆ |  | ☆☆☆ | 6 |
| Ichikawa.T. 2019 | ☆☆☆ |  | ☆☆☆ | 6 |
| Cheng.2018 | ☆☆☆ | ☆ | ☆☆☆ | 7 |
| Cheng. 2019 | ☆☆☆ | ☆ | ☆☆☆ | 7 |
| Gilmar.2018 | ☆☆☆ |  | ☆☆☆ | 6 |
| Inoue etl. 2018 | ☆☆☆ | ☆ | ☆☆☆ | 7 |
| Gitto. 2018 | ☆☆☆ | ☆ | ☆☆☆ | 7 |
| El Sagheerl. 2018 | ☆☆☆ | ☆ | ☆☆☆ | 7 |
| Chida. 2018 | ☆☆☆ | ☆ | ☆☆☆ | 7 |
| [Andrade](https://pubmed.ncbi.nlm.nih.gov/?term=Andrade+VG&cauthor_id=30043871).2018 | ☆☆☆ |  | ☆☆☆ | 6 |
| Juanbeltz. 2017 | ☆☆☆ |  | ☆☆☆ | 6 |
| Endo. 2017 | ☆☆☆ | ☆ | ☆☆☆ | 7 |
| Pedersen.2016 | ☆☆☆ | ☆ | ☆☆☆ | 7 |
| Shimizul. 2018 | ☆☆☆ |  | ☆☆☆ | 6 |
| Beig.2018 | ☆☆☆ |  | ☆☆☆ | 6 |
| Sunl. 2018 | ☆☆☆ |  | ☆☆☆ | 6 |
| Doyle. 2019 | ☆☆☆ |  | ☆☆☆ | 6 |
| Muñoz.H.2020 | ☆☆☆ |  | ☆☆☆ | 6 |
| Sangineto.2020 | ☆☆☆ |  | ☆☆☆ | 6 |
| Inomata.2021 | ☆☆☆ | ☆ | ☆☆☆ | 7 |
| Graf.2020 | ☆☆☆ |  | ☆☆☆ | 6 |
| Chen.2020 | ☆☆☆ |  | ☆☆☆ | 6 |
| Iossa.2021 | ☆☆☆ | ☆ | ☆☆☆ | 7 |
| Eletreby.2021 | ☆☆☆ |  | ☆☆☆ | 6 |
| Nevola.2020 | ☆☆☆ |  | ☆☆☆ | 6 |
| Joshita.2021 | ☆☆☆ |  | ☆☆☆ | 6 |
| Abdoa.2020 | ☆☆☆ | ☆ | ☆☆☆ | 7 |
| Hino.2021 | ☆☆☆ | ☆ | ☆☆☆ | 7 |
| Anca.2023 | ☆☆☆ | ☆ | ☆☆☆ | 7 |
| Ahmed.2023 | ☆☆☆ | ☆ | ☆☆☆ | 7 |
| Diego.2023 | ☆☆☆ |  | ☆☆☆ | 6 |

Supplementary Table 2. The results of Meta analysis

|  | | WMD | 95%CI | | P-value | I^2^ | Publication　Bias | |
| --- | --- | --- | --- | --- | --- | --- | --- | --- |
|  |  |  | L | H |  |  | P | 95%CI |
| SVR（w） | | | | | | | | |
| TG | 12 | 11.353 | -7.772 | 30.479 | 0.245 | 98.2% |  |  |
|  | 24 | -0.033 | -1.357 | 1.292 | 0.961 | 9.2% |  |  |
|  | Over all | 6.112 | -1.470 | 13.694 | 0.114 | 97.8% | 0.396 | -1.498179 3.710265 |
| TC | 12 | 22.743 | 11.064 | 34.423 | 0.000 | 98.2% |  |  |
|  | 24 | 19.401 | 17.335 | 21.468 | 0.000 | 64.7% |  |  |
|  | Over all | 22.207 | 17.149 | 27.265 | 0.000 | 97.7% | 0.523 | -1.688728 3.281656 |
| HDL | 12 | 2.918 | 1.422 | 4.414 | 0.000 | 83.6% |  |  |
|  | 24 | 0.770 | -0.547 | 2.087 | 0.252 | 76.5% |  |  |
|  | Over all | 2.397 | 1.308 | 3.485 | 0.000 | 86.6% | 0.001 | 0.742835 2.671053 |
| LDL | 12 | 19.612 | 12.253 | 26.971 | 0.000 | 97.5% |  |  |
|  | 24 | 17.017 | 13.735 | 20.298 | 0.000 | 92.7% |  |  |
|  | Over all | 19.078 | 15.424 | 22.732 | 0.000 | 97.0% | 0.320 | -1.079881 3.247367 |
| follow-up time | | | | | | | | |
| TG | ETO | 3.403 | -15.915 | 22.721 | 0.730 | 98.9% |  |  |
|  | 12w | 7.616 | -12.893 | 28.124 | 0.467 | 98.2% |  |  |
|  | 24w | -0.772 | -2.170 | 0.626 | 0.279 | 0.0% |  |  |
|  | 1year | 12.243 | 4.131 | 20.355 | 0.003 | 0.0% |  |  |
|  | Over all | 6.112 | -1.470 | 13.694 | 0.114 | 97.9% | 0.396 | -1.498179 3.710265 |
| TC | ETO | 18.905 | 3.495 | 34.314 | 0.016 | 98.4% |  |  |
|  | 4w | 20.901 | 15.335 | 26.468 | 0.000 | 11.9% |  |  |
|  | 12w | 23.255 | 9.414 | 37.096 | 0.001 | 98.3% |  |  |
|  | 24w | 19.635 | 16.353 | 22.917 | 0.000 | 76.0% |  |  |
|  | 1year | 24.900 | 13.669 | 36.131 | 0.000 | 70.4% |  |  |
|  | Over all | 22.207 | 17.149 | 27.265 | 0.000 | 97.7% | 0.523 | -1.688728 3.281656 |
| HDL | ETO | -0.030 | -1.595 | 1.536 | 0.970 | 70.1% |  |  |
|  | 4w | 6.665 | 3.906 | 9.424 | 0.000 | 0.0% |  |  |
|  | 12w | 3.402 | 1.361 | 5.443 | 0.000 | 87.2% |  |  |
|  | 24w | 3.159 | 0.176 | 6.142 | 0.038 | 81.5% |  |  |
|  | 1year | 0.136 | -2.929 | 3.200 | 0.931 | 56.4% |  |  |
|  | Over all | 2.397 | 1.308 | 3.485 | 0.000 | 86.6% | 0.001 | 0.74835 2.671053 |
| LDL | ETO | 16.880 | 4.564 | 29.195 | 0.007 | 98.0% |  |  |
|  | 4w | 14.122 | 6.523 | 21.720 | 0.000 | 65.7% |  |  |
|  | 12w | 20.874 | 11.984 | 29.801 | 0.000 | 97.6% |  |  |
|  | 24w | 17.979 | 14.157 | 21.801 | 0.000 | 89.5% |  |  |
|  | 1year | 19.078 | 10.152 | 24.592 | 0.000 | 77.0% |  |  |
|  | Over all | 19.078 | 15.424 | 22.732 | 0.000 | 97.0% | 0.320 | -1.079881 3.247367 |
| Genotype | | | | | | | | |
| TG | G1b | -1.601 | -7.605 | 4.403 | 0.601 | 0.0% |  |  |
|  | G1 | -5.230 | -20.220 | 9.760 | 0.494 | 87.7% |  |  |
|  | G2 | -0.646 | -14.469 | 13.178 | 0.927 | 0.0% |  |  |
|  | G3 | -4.401 | -20.397 | 11.594 | 0.590 | 0.0% |  |  |
|  | Over all | -2.135 | -7.054 | 2.783 | 0.395 | 30.8% | 0.107 | -0.3558768 3.250818 |
| TC | G1b | 20.947 | 18.467 | 23.428 | 0.000 | 41.8% |  |  |
|  | G1 | 25.952 | 16.413 | 35.492 | 0.000 | 0.0% |  |  |
|  | G2 | 9.290 | 1.937 | 16.642 | 0.013 | 7.8% |  |  |
|  | G3 | 22.646 | 12.647 | 32.646 | 0.000 | 0.0% |  |  |
|  | Over all | 20.236 | 18.011 | 22.461 | 0.000 | 42.1% | 0.605 | -1.248238 2.088183 |
| HDL | G1b | 4.686 | 3.451 | 5.922 | 0.000 | 0.0% |  |  |
|  | G1 | 5.800 | -0.714 | 12.314 | 0.081 | 0.0% |  |  |
|  | G2 | 5.563 | 1.862 | 9.264 | 0.003 | 25.7% |  |  |
|  | G3 | 3.935 | 0.208 | 7.663 | 0.039 | 19.7% |  |  |
|  | Over all | 4.730 | 3.628 | 5.832 | 0.000 | 0.0% | 0.509 | -0.7254048 1.413573 |
| LDL | G1b | 14.801 | 12.657 | 16.946 | 0.000 | 57.0% |  |  |
|  | G1 | 17.300 | 6.161 | 28.439 | 0.002 | 4.0% |  |  |
|  | G2 | 10.519 | 4.068 | 16.971 | 0.001 | 27.2% |  |  |
|  | G3 | 17.762 | 7.770 | 27.754 | 0.000 | 0.0% |  |  |
|  | Over all | 14.597 | 12.634 | 16.560 | 0.000 | 44.2% | 0.097 | -0.2722536 3.023734 |
| ALT | | -40.820 | -49.872 | -31.767 | 0.000 | 93.8% | 0.000 | -7.756814 -3.228709 |
| AST | | -27.339 | -35.294 | -20.875 | 0.000 | 88.2% | 0.011 | -6.653763 -1.116305 |
| TG | Cirrhotics | -9.268 | -19.189 | 0.653 | 0.703 | 0.0% |  |  |
|  | Non-cirrhotics | -1.614 | -9.917 | 6.690 | 0.067 | 0.0% |  |  |
|  | Over all | -4.767 | -11.134 | 1.601 | 0.142 | 0.0% | 0.584 | -1.475874 0.878198 |
| TC | Cirrhotics | 13.824 | 7.310 | 20.337 | 0.000 | 37.2% |  |  |
|  | Non-cirrhotics | 17.139 | 10.601 | 23.676 | 0.000 | 27.4% |  |  |
|  | Over all | 15.475 | 10.861 | 20.089 | 0.000 | 28.3% | 0.633 | -2.564682 1.635233 |
| HDL | Cirrhotics | 1.307 | -0.709 | 3.322 | 0.056 | 0.0% |  |  |
|  | Non-cirrhotics | 1.911 | -0.053 | 3.874 | 0.204 | 41.5% |  |  |
|  | Over all | 1.617 | 0.210 | 3.023 | 0.024 | 16.2% | 0.485 | -1.56898 0.798369 |
| LDL | Cirrhotics | 8.498 | 3.474 | 13.522 | 0.001 | 0.0% |  |  |
|  | Non-cirrhotics | 17.702 | 12.349 | 23.054 | 0.000 | 13.7% |  |  |
|  | Over all | 12.808 | 9.145 | 16.471 | 0.000 | 19.1% | 0.186 | -2.84478  0.6308114 |
