# Supplementary material for: Effects of sustained viral response on lipid in Hepatitis C: a systematic review and meta-analysis
Source: Lipids Health Dis. 2024 Mar 9;23:74. doi: 10.1186/s12944-023-01957-2 (PMC10924993; doi:10.1186/s12944-023-01957-2)
Supplement: Supplementary file 2 — Supplementary Material 2 [file 12944_2023_1957_MOESM2_ESM.pdf]

TingtingMei_Effects_of_sustained_viral_response_on_lipid_in_hepatitis_C_a_systematic_review_and_meta_analysis

WORD COUNT	7171	TIME SUBMITTED	26-OCT-2023 05:15PM
		PAPER ID	103745505

Effects of sustained viral response on lipid in hepatitis C：a systematic review and meta-analysis

Abstract

Background: Direct-acting Antiviral Agents (DAAs) influence the serum lipids of patients with Hepatitis C virus (HCV). This paper presents an analysis of the relevant literature to investigate the effects of DAAs in treating hepatitis C to achieve a sustained viral response (SVR) on lipid parameters.

Methods: PubMed, Web of science, Embase and Central databases were searched, with a deadline of September 2023. Studies on the effects of sustained viral response on lipid parameters after DAAs treatment for hepatitis C were selected. The required information was extracted from the included studies, and then the Stata 12.0 was used to analyze the data quantitatively.

Results: Of 32 studies, the results showed that total cholesterol (TC) levels increased from the end of treatment (WMD=20.144, 95%CI =3.404, 36.884, *P*=0.018) to one year after treatment (WMD=24.900, 95%CI=13.669, 36.131, *P*<0.001). From the end of treatment (WMD=17.728, 95%CI=4.375, 31.082, *P*=0.009) to one year after treatment (WMD=18.528, 95%CI=7.622, 29.433, *P*< 0.001), the levels of low-density lipoprotein (LDL) were also increased. High-density lipoprotein (HDL) levels were elevated from 4 weeks after treatment (WMD=6.665, 95%CI =3.906, 9.424, *P*< 0.001) to 24 weeks after treatment (WMD=3.159,95% CI=0.176, 6.142, *P*=0.038). Triglyceride (TG) levels showed no significant change after the treatment.

Conclusions: Hepatitis C patients who achieved SVR on DAAs showed the increase of lipid levels and the improvement of hepatic inflammation indicators AST and ALT. This may provide evidence-based medical evidence for the follow-up and monitoring of blood lipids and hyperlipidemia treatment.

Registration: PROSPERO CRD42020180793.

Keywords: Direct-acting Antiviral Agents; hepatitis C; Sustained Viral Response; lipid; Meta-analysis.

Background

Hepatitis C virus (HCV) is a plus-strand RNA virus whose infection is mainly confined to liver cells and is an important cause of cirrhosis, liver cancer, and liver transplantation [1]. In 2019, the World Health Organization reported that about 58 million people worldwide are infected with chronic hepatitis C [2].

Studies have found that infection with hepatitis C virus can affect lipid and lipoprotein metabolism levels in the body [3]. The typical presentation is enhanced lipid production and decreased lipoprotein secretion, which accelerates the process of atherosclerosis and liver steatosis [4,5]. Moreover, clinical studies have found that the prevalence of liver steatosis in CHC patients is 40-86%, which is much higher than 20-50% of other chronic liver disease patients without hcv infection [6,7]. Therefore treatment of HCV may be crucial in regulating the lipid metabolic disorders it causes. Sustained viral response (SVR) is defined as the disappearance of HCV RNA in

plasma at 12 or 24 weeks after completion of treatment [8]. Before the advent of direct-acting antiviral drugs (DAAs), hepatitis C was mainly based on interferon (IFN), but its SVR rate was only about 50%, with serious side effects [9]. Recently developed DAAs are emerging as a new branch of standard HCV therapy that can significantly improve treatment outcomes [3]. Scott A McDonald et al [10]. compared and analyzed decompensated cirrhosis patients who received interferon-free therapies in the pre-DAA era with those who received interferon-free DAA therapies in the DAA era. The risk of liver-related death in patients with decompensated cirrhosis in the DAA era was significantly lower than that in the pre-DAA era. In addition, Tanaka et al [11]. found that DAA administration after hepatectomy could improve liver function in patients with HCC, which may prolong postoperative survival. Moreover, the study also found that DAA can adversely affect lipid profiles by eradicating HCV, which increases the risk of cardiovascular disease development. However, SVR can ultimately improve overall cardiovascular mortality by eliminating many other harmful effects of HCV [12].Therefore, it is particularly important to further understand the influence of DAAs treatment on the lipid profile of patients who achieved a sustained viral response to HCV.

Currently, there are many domestic and foreign studies on the relationship between DAAs treatment and lipid parameters in patients with hepatitis C. Still, the results of each study are not identical. Kawagishi N et al [13]. In 2018, the successful elimination of HCV with interferon-free DAAs therapy reduced low-density lipoprotein cholesterol (LDL-C) levels in patients with higher baseline values and in

patients with hepatic steatosis and dyslipidemia in SVR24. Increased LDL-C levels are accompanied by increased sdLDL-C (Small and dense LDL-C) levels. However, Pedersen et al. [14] found that successful DAA treatment could increase LDL and High-density lipoprotein (HDL). In contrast, Triglyceride (TG) levels were reduced after treatment. To unify these differences, we performed a meta-analysis to comprehensively evaluate the effects of persistent hepatitis C viral response on lipid parameters. In particular, the duration of lipid changes and the changes of lipid in patients with different genotypes or different SVR. So as to provide a reference for clinicians to individualized treatment.

Materials and Methods

This meta-analysis followed the PRISMA guidelines [15]. The search strategies and inclusion and exclusion criteria were registered with PROSPERO (PROSPEROCRD 42020180793).

Search strategies

The Medical Subject Heading terms and keywords used in the search process mainly included: “Hepatitis C,” “Hepacivirus,” “Sofosbuvir,” “DAA,” “Lipid Metabolism,” “Cholesterol,” “Triglyceride,” “Cholesterol, HDL,” “Cholesterol, LDL,” and “Apolipoproteins.” The databases searched included PubMed, Central, Embase, Web of Science. The search period was September 2023.

Inclusion and exclusion criteria

The inclusion criteria:(1) The subjects of studies were patients with HCV; (2) studies on HCV patients who received DAA therapy; (3) availability of relevant lipid data before and after treatment; (4) studies in which persistent viral responses in HCV patients were clear and (5) prospective or retrospective studies. The exclusion criteria: (1) the study population was co-infected with HIV/HCV; (2) interferon was included in the treatment regimen; (3) text type: reviews, editorials, letters, case reports, personal newsletters, pre-prints and abstracts. Literature screening and data extraction were conducted independently by two researchers. First, primary literature retrieval was carried out, and then the literature retrieved at the primary level was screened according to pre-set criteria. When two researchers had different opinions, they discussed and settled together to reach a unified standard.

Quality evaluation of literature evidence

The Newcastle Ottawa Scale (NOS) was used to assess study quality. We assessed the quality of the evidence for each relevant study. (The total score is 9 points. 7-9points：High-quality; 4-6 points：Moderate-quality; 0-3 points：Low-quality)

Data extraction

Two independent researchers conducted the data extraction according to the formulated unified and standardized data tables. Relevant experts were invited to review these controversial issues. Data were extracted based on the following parameters: First author, Publication year, Country or region, Study type, Age,

Percentage of males, DAAs protocol, Genotype, Sample size, SVR status, Baseline lipid parameters, and Lipid parameters at and after treatment (12 weeks, 24 weeks or one year). Mean ± standard deviation (SD) or median (interquartile spacing) was used to express age parameters and sex information was expressed as the percentage of males in each group.

The primary outcome

(1) Changes in TG, total cholesterol (TC), LDL and HDL levels after DAAs treatment (at the end treatment, 4w after treatment, 12w after treatment, 24w after treatment, and one year after treatment) in patients who achieved sustained viral response compared with baseline; (2) Changes in lipids of patients with different SVR (SVR12 or SVR24) or different genotypes; (3) Changes of indicators of hepatic inflammation (AST and ALT) before and after treatments; (4) In part of the study, changes of lipids in patients with cirrhosis and non-cirrhosis were analyzed.

Statistical analysis

The software used in this study was Stata 12.0, and WMD values and the corresponding 95% confidence interval (CI) were used to measure and evaluate the association strength. Heterogeneity between studies was tested using the Q-test statistics and I^2 values, and the I^2 value was used to measure heterogeneity. Heterogeneity test $I^2 < 50\%$ indicated no significant heterogeneity. Therefore, WMD was calculated using a fixed effects model. If heterogeneity was present, a

random-effects model was selected. The Z statistic was used to test the combined WMD values. Differences with statistical significance was defined as $P<0.05$. Egger's linear regression method was used to evaluate publication bias. Sensitivity analysis was performed by eliminating individual studies individually.

Results

Search results

A total of 1159 qualified studies were preliminarily retrieved according to the set retrieval formula, and 933 articles were retrieved that might be included in the study excluding 226 duplicate studies. After reading titles and abstracts, 848 irrelevant papers were excluded. 85 studies were excluded after reading the full text. Finally, 29 eligible papers were included **(Figure 1).**

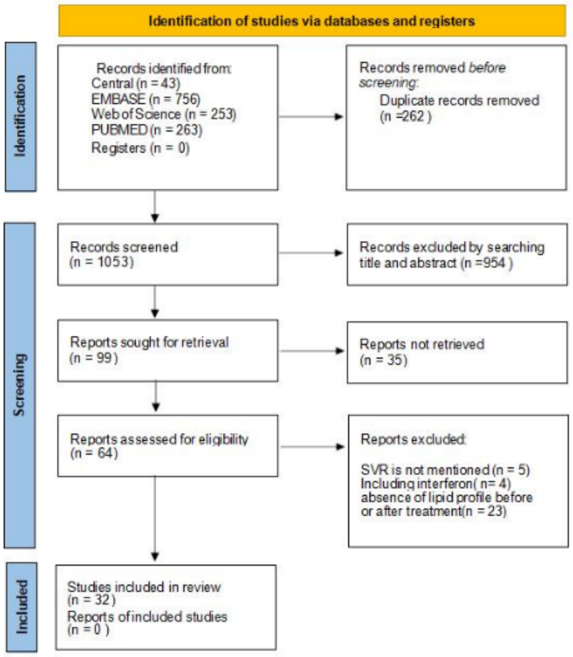

Figure 1. Flow diagram of study selection.

Basic features of the included studies

Eleven studies were prospective studies, 13 were retrospective studies, one was retro-prospective study and seven types of studies were not mentioned in this meta-analysis; patients achieved SVR12 in twenty five studies and SVR24 in seven studies; one study reported changes in lipids in patients with significant and non-significant liver fibrosis, and three studies reported changes in lipids in patients with cirrhosis and non-cirrhosis. The research areas include Asia (India, Japan, Tai Wan), South America (Brazil), Europe (Italy, Spain, Germany), Africa (Egypt), North America (America, Canada) and Oceania (New Zealand). The basic characteristics are summarized in **Table 1**.

Table 1. Characteristics of Studies and patients

First author Year	Country / Region	Study design	Antiviral regimens	Genotype	Weeks of treatment	SVR (w)	Number	Age (Years)	Males
Jain [16] 2018	India	prospective	SOF/DCV	G3	12	SVR12	47	38 ± 13	60%
Ichikawa[17] 2019	Japan	retrospective	DCV/ASV	G1b	24	SVR24	38	70.92±11.02	36%
Ichikawa [18] 2019	Japan	retrospective	Multiple DAA regimens	Multiple genotypes	12/24	SVR12	48	70.1±11.08	42%
Cheng [19] 2018	Tai wan	prospective	SOF/DCV	G2	12	SVR12	31	65.0±13.2	28.10%
Cheng [20] 2019	Tai wan	prospective	Multiple DAA regimens	Multiple genotypes	12/24	SVR12	102<br>AF 76<br>NAF 26	66.0±10.7<br>68.0±9.3<br>60.3±12.6	33.30%<br>32.90%<br>34.6%
Gilmar [21] 2018	Brazil	retrospective	Multiple DAA regimens	Multiple genotypes	12/24	SVR12	43	60.5 ± 9.5	27.90%
Inoue [22] 2018	Japan	NA	DCV/ASV<br>SOF/LDV<br>SOF/RBV	G1b<br>G1b<br>G2	24<br>12<br>12	SVR24<br>SVR24<br>SVR24	69<br>84<br>45	68.3±10.5<br>64.4±13.3<br>62.0±15.3	38.80%<br>43.50%<br>32.60%
Gitto [23] 2018	Italy	NA	Multiple DAA regimens	Multiple genotypes	12/24	SVR24	93	64 ± 12	60.20%
El Sagheer [24] 2018	Egypt	NA	SIM/SOF	G4	12	SVR12	79	47 ± 12	58.80%
Chida [25] 2018	America	retrospective	DCV/ASV	G1b	24	SVR12	67	71±9	40.00%
Andrade [26] 2018	Brazil	retro-prospective	Multiple DAA regimens	Multiple genotypes	12	SVR12	95	56 ± 9	70%
Juanbeltz [27] 2017	Spain	retrospective	Multiple DAA regimens	Multiple genotypes	12/16/24	SVR12	212	53.6 ± 9.3	71.80%
Endo [28] 2017	Japan	NA	DCV/ASV<br>SOF/LDV	G1b<br>G1b	24<br>24	SVR12<br>SVR12	121<br>132	68.4 ± 11.8<br>66.7 ± 13.1	48.80%<br>37.10%
Pedersen [14] 2016	America	prospective	SOF/RBV<br>SOF/RBV	G2<br>G3	12/24<br>12/24	SVR12<br>SVR12	58<br>C 33<br>NC 25<br>31<br>C 14<br>NC 17	55.5 ± 12.1<br>54.6 ± 10.8	61.20%<br>64.50%
Shimizu [29] 2018	Japan	NA	Multiple DAA regimens	G1/G2	12/24	SVR12	70	66 (59–73)	41.40%
Beig [30] 2018	New Zealand	retrospective	Multiple DAA regimens	NA	NA	SVR12	35	NA	NA

Sun [31] 2017	Tai wan	NA	GZR/EBV or SOF/LDV	G1	12	SVR12	22	60 (39~83)	50%
Doyle [32] 2019	Canada	NA	PrOD	G1a/G1b	12	SVR12	23	54±11.6	71%
Muñoz.H [33] 2020	NA	prospective	Multiple DAA regimens	Multiple genotypes	12	SVR12	109	53.6±10.8	69.70%
Sangineto [34] 2021	Italy	retrospective	Multiple DAA regimens	NA	NA	SVR24	95	67.1 ± 0.8	50.60%
Inomata [35] 2022	Japan	retrospective	SOF/LDV	G1b	12	SVR12	22	60.5 (55-69)	50%
Graf [36] 2020	Germany	retrospective	Multiple DAA regimens	Multiple genotypes	12	SVR24	45	51.7 ± 14.1	47.80%
Chen [37] 2020	Tai wan	prospective	Multiple DAA regimens	Multiple genotypes	12/24	SVR12	102	65.9 ± 9.9	32.40%
Iossa [38] 2021	Italy	retrospective	Multiple DAA regimens	Multiple genotypes	12	SVR24	47<br>C 31<br>NC 18	66 (62–71)	42.90%
Eletreby [39] 2021	Egypt	prospective	SOF/DAC ± RBV	Multiple genotypes	12/24	SVR12	264	51.73±10.24	89.20%
Nevola [40] 2020	Italy	prospective	Multiple DAA regimens	Multiple genotypes	8-24	SVR24	243	68 (62–74)	46.90%
Joshita [41] 2021	Japan	retrospective	Multiple DAA regimens	Multiple genotypes	8/12/24	SVR12	231	70 (63–76)	42.00%
Abdoa [42] 2020	Egypt	retrospective	SOF/DCV	NA	12/24	SVR12	98<br>C 32<br>NC 66	51.54 ±6.91	44.90%
Hino [43] 2021	NA	retrospective	Multiple DAA regimens	Multiple genotypes	12	SVR12	67	70.0 (62-77)	40.30%
Anca [44] 2023	Italy	retrospective	Multiple DAA regimens	NA	12	SVR12	132	61.17 ± 9.11	35.6%
Ahmed[45] 2023	Egypt.	retrospective	Multiple DAA regimens	NA	12	SVR12	100	50.99 ± 8.75	100%
Diego[46] 2023	Spain	retrospective	Multiple DAA regimens	Multiple genotypes	8/12	SVR12	83	55 (49-63)	49.4%

NA,not available; AF,Advanced fibrosis; NAF,Non-advanced fibrosis;C,Cirrhotics; NC,Non-cirrhotics;

SOF: Sofosbuvir; DCV: Daclatasvir;ASV: Asunaprevir;LDV: Ledipasvir;

LDV: Ledipasvir;RBV: Ribavirin;SIM: Simeprevir;GZR: Grazoprevir;

EBV: Elbasvir; PrOD: paritaprevir/ritonavir/ombitasvir/dasabuvir;DAC: Daclatasvir

Quality evaluation of literature evidence

NOS scores were performed on the 32 included studies , and the results showed that the scores of all studies were no less than six, indicating that the 32 studies were of medium and high quality. **(Supplementary Table 1)**

Change in TC after antiviral therapy

Figure 2 shows the change in total cholesterol levels in patients who achieved a sustained viral response after DAAs treatment. Results show that the TC level increased at the end of treatment (WMD= 18.905, 95%CI=3.495, 34.314, *P*=0.016), 4 weeks after completion of treatment (WMD=20.901, 95%CI=15.335, 26.468, *P*<0.001), 12 weeks (WMD= 23.255, 95%CI=9.414, 37.096, *P*=0.001), 24 weeks (WMD=19.635, 95% CI= 16.353, 22.917, *P*< 0.001) and one year (WMD=24.900， 95% CI=13.669, 36.131， *P*<0.001) after treatment compared with that before the treatment. Because of the high heterogeneity of the study, sensitivity analyses were performed and the results were found to be stable; there was no significant publication bias, and the random effects model was used for statistical analysis.

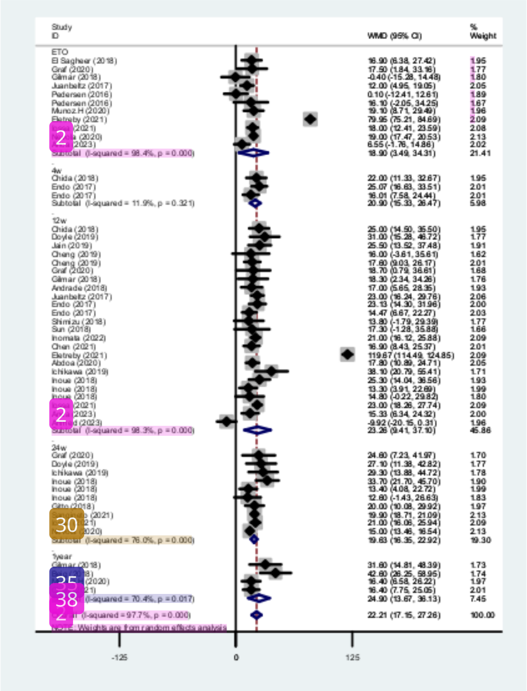

Figure 2 Changes of serum TC after treatment. Forest plots with weighted mean difference (WMD) and 95% confidence interval (CI) at each time point.

Change in LDL after antiviral therapy

Figure 3 shows the changes in LDL levels in patients who achieved a sustained viral response after DAAs treatment ended. Compared with before of treatment, serum LDL levels increased from the end of treatment (WMD=16.88, 95%CI = 4.564, 29.195, P=0.007) to 1 year after treatment (WMD= 17.372, 95%CI=10.152, 24.592, P<0.001). Heterogeneity was the same as that for TC, and there was no significant publication bias.

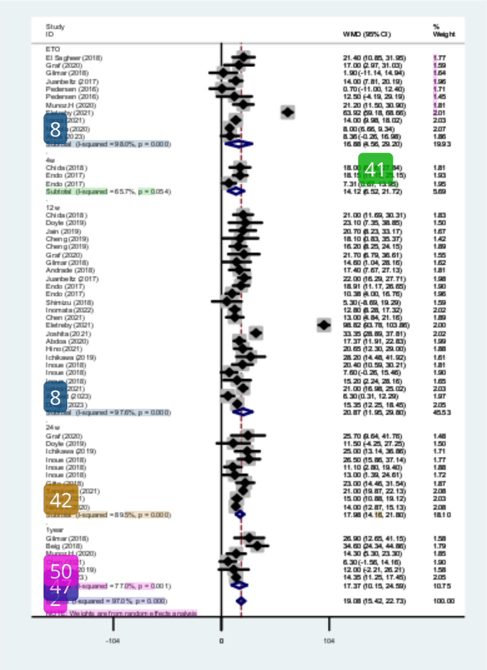

Figure 3 Changes of serum LDL after treatment. Forest plots with weighted mean difference (WMD) and 95% confidence interval (CI) at each time point.

Change in HDL after antiviral therapy

As Figure 4 shows, the levels of HDL from 4 weeks after treatment (WMD=6.665, 95%CI =3.906-9.424, *P*< 0.001) to 24 weeks after treatment (WMD=3.159, 95%CI =0.176-6.142, *P*=0.038) were elevated compared with that before the treatment. However, at the end of treatment (WMD=-0.030, 95%CI =-1.595-1.536, ***P***=0.970) and one year after the end of treatment (WMD=0.136, 95%CI = -2.929-3.200, *P*=0.931), the changes in HDL levels were not statistically significant.

The results showed a P value <0.05 for publication bias, so a trim and fill analysis was carried out. Before the trim-and-fill test, the heterogeneity test was Q= 381.642, $P < 0.001$, and the random effect model was adopted. The combined effect size result was 2.397, 95% CI=1.308-3.485, and the heterogeneity test after the trim-and-fill Q= 718.837, $P < 0.001$.The combined effect size was 0.844 (with 95% CI=0.270-2.644) . The results were not reversed before and after trim-and-fill analysis, indicating that the results were relatively robust.

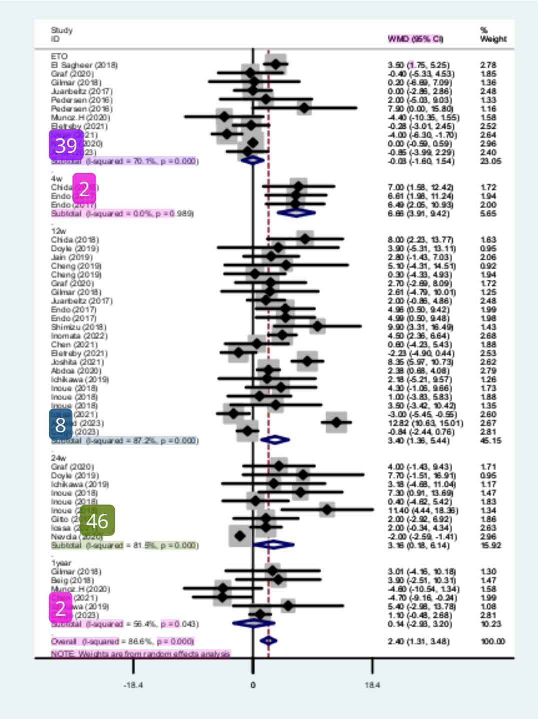

Figure 4 Changes of serum HDL after treatment.Forest plots with weighted mean difference (WMD) and 95% confidence interval (CI) at each time point.

Change in TG after antiviral therapy

Figure 5 shows the changes of TG in patients before and after treatment, and there was no statistically significant at the end of treatment (WMD= 3.403, 95%CI =-15.915-22.721, *P*= 0.730), 12 weeks after completion of treatment (WMD=7.616, 95%CI =-12.893, 28.1248, *P*=0.467), 24 weeks after completion of treatment (WMD= -0.772,95% CI=-2.170,0.626, *P*=0.279)

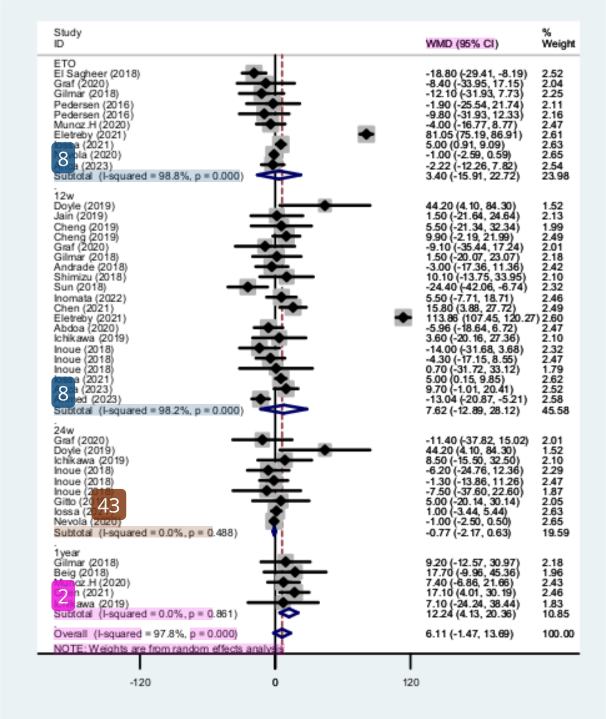

Figure5 Changes of serumTG after treatment. Forest plots with weighted mean difference (WMD) and 95% confidence interval (CI) at each time point.

Subgroup analysis was performed according to different SVR

Supplementary Figure 1 and 2 show the changes in TC and LDL levels in patients with different SVR. The results showed that in patients who achieved SVR12 and

SVR24,the serum TC levels (SVR12: WMD=22.743, 95%CI =11.064, 34.423, $P< 0.001$; SVR24: WMD=19.401, 95% CI=17.335,21.468, $P< 0.001$;) and LDL levels (SVR12: WMD=19.612, 95%CI =12.253, 26.971, $P< 0.001$; SVR24: WMD=17.017, 95% CI=13.735,20.298, $P< 0.001$;) increased after treatment. There was no publication bias.

Subgroup analysis was performed according to different genotypes

Supplementary Figure 3 shows the changes in TC and LDL levels in the patients with different genotypes. The results showed that serum TC and LDL levels of patients with the G1b, G1, G2, and G3 genotypes increased after treatment, with no publication bias. (**Supplementary Table 2**).

Change of AST and ALT

Seven studies reported changes in AST levels before and after treatment. AST levels decreased after treatment compared to before treatment (WMD=-27.339, 95%CI = -35.294, -19.385, $P< 0.001$), as shown in Supplementary Figure 4. Ten studies reported changes in ALT before and after treatment, and ALT levels also decreased after treatment (WMD=-40.820, 95%CI = -49.872, -31.767, $P< 0.001$), as shown in Supplementary Figure 5. There was a publication bias in both cases. After two iterations, there were no missing articles, and the research results were relatively reliable.

Change in lipids in patients with cirrhosis and non-cirrhosis

Three studies reported changes in lipid levels in patients with cirrhosis and non-cirrhosis. Pooled analysis showed that TC in cirrhosis and non-cirrhosis patients (cirrhosis: WMD=13.824, 95% CI=7.310, 20.337, P< 0.001; non-cirrhosis: WMD=17.139, 95%CI= 10.601,23.676, P< 0.001) and LDL (cirrhosis: WMD=8.498, 95%CI =3.474, 13.522, P= 0.001;non-cirrhosis: WMD=17.702, 95%CI =12.349, 23.67623.054, P< 0.001) after treatment was increased compared with that before treatment, as shown in Supplementary Figure 6 and 7. TG and HDL levels were not statistically significant in either population, and there was no significant heterogeneity or publication bias.

Discussion

This study was conducted on lipid changes in patients with HCV who achieved SVR after DAA treatment. In addition, the long-term effect of DAA treatment on blood lipid levels were studied.

Currently, the mechanism of the interaction and influence between HCV and blood lipids remains unclear. Previous studies have shown that inhibition of cholesteryl ester and triglyceride synthesis can inhibit viral infection by inhibiting the assembly process of hepatitis C virus [47].On the other hand, hepatitis C virus itself is a high-fat lipovirus particle, very similar to very low-density lipoprotein(VLDL), which can alter liver lipoprotein-related functions in a variety of ways, including by impairing the VLDL release pathway. Therefore, it is related to the accumulation of liver lipids and the pathogenesis of dyslipidemia [48]. Additionally, it can enhance

replication by regulating host cell lipid metabolism [49]. In patients with hepatitis C who have achieved SVR, HCV RNA cannot be detected in the plasma, which may reduce lipid metabolism in the host and affect the patient's lipid levels. Currently, there are many treatments for HCV infection, but compared with traditional peginterferon-based treatment regimens, recent DAAs have increased the persistent viral response rate in patients with chronic HCV infection [50]. Recent studies have shown that DAA treatment for HCV is associated with favorable cardiovascular outcomes [51]. In addition, HCV treatment reduces the risk of insulin resistance and diabetes [52,53]. There have been many studies on HCV treatment and lipid changes, but the results have not been the same. Stefan et al.[54] found that suppression and elimination of HCV by DAAs without interferon had no effect on TG but increased TC levels; however, interferon-based therapy increased TG and TC during treatment and led to elevated TC when a sustained virological response was achieved. DAAs therapy for hepatitis C is closely related to lipid changes in patients [55].

In 2021, RosannaVillani et al [56]. conducted a meta-analysis in which they examined changes in blood lipid levels during DAA treatment and at 12 and 24 weeks after the end of treatment. The results showed that the patient's TC, LDL, and HDL levels increased, which persisted for 24 weeks after the end of treatment. The differences between this study and that of RosannaVillani et al are as follows:1. Study population: the study population of Rosanna Villani et al. included patients treated with DAA, while this study targeted patients who achieved SVR after DAA treatment, which can exclude the effect of SVR on patients' blood lipids; 2. Observation time: R

et al. analyzed the changes in blood lipids of patients from the treatment period to 24 weeks after treatment; this study was extended to 1 year after the end of treatment to observe further the long-term effects of DAA on patients' blood lipids. 3. Subgroup analysis: Further subgroup analysis was performed for different sustained viral response times and different genotypes in this study. 4. The meta-analysis included many number of studies, and the results were more robust.

Finally, 32 articles were included in this meta-analysis, and there was a correlation between persistent hepatitis C virus response and lipid changes. Serum TC and LDL levels increased from the end of treatment to one year after the end of treatment. Serum HDL levels also increased from 4 to 24 weeks after the end of treatment, but TG did not change significantly. In addition, the results of hepatic inflammation analysis showed that AST and ALT levels improved after treatment. It can be seen that after hepatitis C patients achieve SVR by DAA treatment, the main changes in blood lipids are persistent increases in TC, LDL and HDL, while TG has no significant change. This may provide a reference for the treatment of lipid-lowering in patients with hepatitis C and the long-term detection of lipids.

Hepatitis C has a variety of genotypes, in this study, by analyzing the changes of lipids in patients with different genotypes who obtained SVR, it can be found that the levels of serum TC and LDL in patients with G1b, G1, G2 and G3 genotypes were all increased after treatment. Similarly, Jain et al.[57] demonstrated a significant increase in TC and LDL with SVR in HCV genotype 3 patients. And Doyle et al.[58] also

found a significant increase in TC and LDL levels after achieving SVR in a study of genotype 1 patients. Antiviral therapy affects lipid metabolism [59], and the differences in the effects of different genotypes on blood lipids in patients with SVR seem to be inconclusive at present, which may require further research in the future.

In addition, a sensitivity analysis was conducted by excluding single studies. The sensitivity analysis did not affect the combined effect size by excluding single studies, suggesting that the results of the meta-analysis were robust.

Strengths and limitations

This study comprehensively analyzed the lipid changes in patients who achieved SVR after DAA treatment. The changes of lipid in patients with different genotypes and different SVR were also compared.

In addition, this study had some limitations. First, only English databases were selected for literature retrieval; Therefore, the scope of the selected literature was not wide enough, and the number of included studies was small. Further high-quality studies with larger samples size are required. Second, in some studies of this study showed significant heterogeneity. Although sensitivity analysis was conducted and a random effects model was finally adopted for analysis, the stability of the meta-analysis results may be affected to a certain extent. Third, there was a publication bias in part of the analysis process, which was identified and processed.

Fourth, the age of the research population included in the literature is between 50 and 70 years, and more studies on other age groups are needed.

Conclusion

In conclusion, this meta-analysis showed that the sustained viral response to DAAs treatment in patients with hepatitis C was correlated with an increase in serum TC, LDL and HDL levels and an improvement in AST and ALT levels after treatment. There were similar changes in serum TC and LDL levels in patients with and without cirrhosis. which provided a reference value for long-term lipid-lowering therapy in patients. Future research may focus on these changes and the choice of lipid-lowering therapy to reduce the risk of cardiovascular disease in patients.

Abbreviations

DAAs: Direct-acting Antiviral drugs; HCV: Hepatitis C virus; SVR: Sustained Viral Response; TC: Total cholesterol; LDL: Low-density lipoprotein; HDL: High-density lipoprotein; TG: Triglyceride; IFN: interferon; NOS: Newcastle Ottawa Scale; NA: Not available; AF: Advanced fibrosis; NAF: Non-advanced fibrosis; C: Cirrhotics; NC: Non-cirrhotics; SOF: Sofosbuvir; DCV: Daclatasvir; ASV: Asunaprevir; LDV: Ledipasvir; LDV: Ledipasvir; RBV: Ribavirin; SIM: Simeprevir; GZR: Grazoprevir; EBV: Elbasvir; PrOD: paritaprevir/ritonavir/ombitasvir/dasabuvir; DAC: Daclatasvir.

TingtingMei_Effects_of_sustained_viral_response_on_lipid_...

ORIGINALITY REPORT

14%
SIMILARITY INDEX

PRIMARY SOURCES

1	"Posters (Abstracts 264-2239)", Hepatology, 2017<br>Crossref	115 words — 2%
2	downloads.hindawi.com<br>Internet	115 words — 2%
3	"Posters (Abstracts 289–2348)", Hepatology, 2019<br>Crossref	64 words — 1%
4	"AASLD Abstracts", Hepatology, 2012.<br>Crossref	55 words — 1%
5	www.researchgate.net<br>Internet	54 words — 1%
6	www.science.gov<br>Internet	54 words — 1%
7	"Abstracts", Hepatology International, 2020<br>Crossref	46 words — 1%
8	www.efsa.europa.eu<br>Internet	40 words — 1%
9	Rosanna Villani, Francesca Di Cosimo, Antonino Davide Romano, Moris Sangineto, Gaetano Serviddio. "Serum lipid profile in HCV patients treated with	34 words — < 1%

direct-acting antivirals: a systematic review and meta-analysis", Scientific Reports, 2021
Crossref

10 pdffox.com 29 words — < 1%
Internet

11 "Abstracts of the 27th Annual Conference of APASL, March 14–18, 2018, New Delhi, India", Hepatology International, 2018 24 words — < 1%
Crossref

12 Despres, J.P.. "Effect of the amount of body fat on the age-associated increase in serum cholesterol", Preventive Medicine, 198807 22 words — < 1%
Crossref

13 "Abstract", Hepatology International, 2008 15 words — < 1%
Crossref

14 jrenhep.com 15 words — < 1%
Internet

15 nepis.epa.gov 14 words — < 1%
Internet

16 cyberleninka.org 13 words — < 1%
Internet

17 link.springer.com 12 words — < 1%
Internet

18 www.dovepress.com 12 words — < 1%
Internet

19 Filomena Morisco, Rocco Granata, Silvia Camera, Antonio Ippolito et al. "Optimization of direct 11 words — < 1%

anti-viral agent treatment schedule: Focus on HCV genotype 3", United European Gastroenterology Journal, 2017
Crossref

20	journals.plos.org<br>Internet	11 words — < 1%
21	pubmed.ncbi.nlm.nih.gov<br>Internet	11 words — < 1%
22	Débora Corrêa Espiña, Fabiano Barbosa Carvalho, Daniela Zanini, Josiane Bizzi Schlemmer et al. "A more accurate profile of Achyrocline satureioides hypocholesterolemic activity", Cell Biochemistry and Function, 2012<br>Crossref	10 words — < 1%
23	jnnp.bmj.com<br>Internet	10 words — < 1%
24	www.frontiersin.org<br>Internet	10 words — < 1%
25	www.ucsf-ahp.org<br>Internet	10 words — < 1%
26	A Mathew. "Chronic kidney disease and postoperative mortality: A systematic review and meta-analysis", Kidney International, 05/2008<br>Crossref	9 words — < 1%
27	journals.lww.com<br>Internet	9 words — < 1%
28	pure.rug.nl<br>Internet	9 words — < 1%

29 rcastoragev2.blob.core.windows.net
Internet
9 words — < 1%

30 9dok.org
Internet
8 words — < 1%

31 Alberto Verrotti, Fania Basciani, Sergio Domizio, Giuseppe Sabatino, Guido Morgese, Francesco Chiarelli. "Serum lipids and lipoproteins in patients treated with antiepileptic drugs", Pediatric Neurology, 1998
Crossref
8 words — < 1%

32 Coilly, Audrey, Bruno Roche, Jean- Charles Duclos-Vallée, and Didier Samuel. "News and challenges in the treatment of hepatitis C in liver transplantation", Liver International, 2016.
Crossref
8 words — < 1%

33 Filippatos, T.D.. "Effects of hormonal treatment on lipids in patients with cancer", Cancer Treatment Reviews, 200904
Crossref
8 words — < 1%

34 Jie Wang, Wenjun You, Zhaohai Jing, Robin Wang, Zhengju Fu, Yangang Wang. "Increased risk of vertebral fracture in patients with diabetes: a meta-analysis of cohort studies", International Orthopaedics, 2016
Crossref
8 words — < 1%

35 Marjorie Chinen, Thomas Hoop, Lorena Alcázar, María Balarin, Josh Sennett. "Vocational and business training to improve women's labour market outcomes in low- and middle-income countries: a systematic review", Campbell Systematic Reviews, 2017
Crossref
8 words — < 1%

36 O. Znoyko, M. Maevskaya, E. Klimova, S. Kizhlo et al. "850 EARLY VIROLOGIC RESPONSE (EVR) IN TREATMENT-NAÏVE MONO-INFECTED CHRONIC HEPATITIS C (CHC) PATIENTS TREATED WITH CEPEGINTERFERON-alfa-2b (cePEG-IFNa-2b) PLUS RIBAVIRIN", Journal of Hepatology, 2013 — 8 words — < 1%
Crossref

37 Shengbing Li. "Association of adipose most abundant transcript 1 gene (apM1) with type 2 diabetes mellitus in a Chinese population: a meta-analysis of case-control studies", Clinical Endocrinology, 6/2008 — 8 words — < 1%
Crossref

38 clok.uclan.ac.uk — 8 words — < 1%
Internet

39 digitalcollections.ohsu.edu — 8 words — < 1%
Internet

40 etheses.bham.ac.uk — 8 words — < 1%
Internet

41 healthdocbox.com — 8 words — < 1%
Internet

42 ijbms.mums.ac.ir — 8 words — < 1%
Internet

43 pdfs.semanticscholar.org — 8 words — < 1%
Internet

44 research-information.bris.ac.uk — 8 words — < 1%
Internet

45 www.jemds.com — 8 words — < 1%
Internet

46	www.medrxiv.org<br>Internet	8 words — < 1%
47	www.rama.mahidol.ac.th<br>Internet	8 words — < 1%
48	Rena Kaneko, Natsuko Nakazaki, Risa Omori, Yuichiro Yano, Masazumi Ogawa, Yuzuru Sato. "Efficacy of direct-acting antiviral treatment for chronic hepatitis C: A single hospital experience", World Journal of Hepatology, 2018<br>Crossref	6 words — < 1%
49	Vikas Singh, Ghanshyambhai T. Savani, Rodrigo Mendirichaga, Anil K. Jonnalagadda, Mauricio G. Cohen, Igor F. Palacios. "Frequency of Complications Including Death from Coronary Artery Bypass Grafting in Patients with Hepatic Cirrhosis", The American Journal of Cardiology, 2018<br>Crossref	6 words — < 1%
50	Yue You, Zhizhen Liu, Yannan Chen, Ying Xu, Jiawei Qin, Shuai Guo, Jia Huang, Jing Tao. "The prevalence of mild cognitive impairment in type 2 diabetes mellitus patients: a systematic review and meta-analysis", Acta Diabetologica, 2021<br>Crossref	6 words — < 1%

EXCLUDE QUOTES	ON	EXCLUDE SOURCES	OFF
EXCLUDE BIBLIOGRAPHY	ON	EXCLUDE MATCHES	OFF
