# Supplementary material for: Effects of sustained viral response on lipid in Hepatitis C: a systematic review and meta-analysis
Source: Lipids Health Dis. 2024 Mar 9;23:74. doi: 10.1186/s12944-023-01957-2 (PMC10924993; doi:10.1186/s12944-023-01957-2)
Supplement: Supplementary file 4 — Supplementary Material 4 [file 12944_2023_1957_MOESM4_ESM.docx]

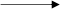

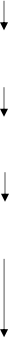

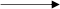

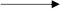

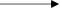


**Identification**

|  |
| --- |

| Records removed *before*  *screening*:  Duplicate records removed (n =262 ) |
| --- |

**Screening**

|  |
| --- |

| Records excluded by searching title and abstract (n =954 ) |
| --- |

| Reports not retrieved  (n = 35) |
| --- |

| Reports excluded:  SVR is not mentioned (n = 5) Including interferon( n= 4) absence of lipid profile before or after treatment(n = 23) |
| --- |

**Included**

|  |
| --- |

| **Identification of studies via databases and registers** |
| --- |

| Records identified from:  Central (n = 43)  EMBASE (n = 756)  Web of Science (n = 253)  PUBMED (n = 263)  Registers (n = 0) |
| --- |

| Records screened  (n = 1053) |
| --- |

| Reports sought for retrieval  (n = 99) |
| --- |

| Reports assessed for eligibility (n = 64) |
| --- |

| Studies included in review  (n = 32)  Reports of included studies  (n = 0 ) |
| --- |
