# Supplementary material for: Effects of sustained viral response on lipid in Hepatitis C: a systematic review and meta-analysis
Source: Lipids Health Dis. 2024 Mar 9;23:74. doi: 10.1186/s12944-023-01957-2 (PMC10924993; doi:10.1186/s12944-023-01957-2)
Supplement: Supplementary file 5 — Supplementary Material 5 [file 12944_2023_1957_MOESM5_ESM.docx]

尊敬的作者：

感谢您选择与Editage合作！

请您在已润色的文件中查看修改和批注。

如果您对润色内容有疑问，或收到期刊审稿人审稿意见，或者希望我们检查您所做的其他修改，请使用您EditageOnline^TM^帐户上的后续支持选项（<http://app.editage.cn/>）。

我们想了解您对我们的服务有什么看法，以及我们如何才能做得更好。请通过您的EditageOnline^TM^帐户将您对服务的反馈发送给我们。

**致谢编辑支持**

不少作者在他们文稿中表达了对Editage编辑支持的感谢。根据著名的国际医学期刊编辑委员会（ICMJE）发表指南中关于作者署名、润色或写作支持， 发表文稿中应给与致谢。这样的致谢也有助于向期刊编辑/审稿人保证，英语已经被彻底审查过，并且符合发表所需的标准。

如果您想致谢我们对这篇论文的编辑支持，您可以在论文的“致谢”部分加上以下句子：*We would like to thank Editage (www.editage.cn) for English language editing.*

致敬

您的编辑

| **Additional notes** |
| --- |

**Formatting**

(To ensure that your paper conforms to the formatting requirements of the target publication)

1. Title page:
2. Author information:
3. Abstract:
4. In-text citations:
5. References:
6. Section headings:

| **Quick tip** |
| --- |
| **Guideline** |
| Avoid Wordiness |
| **Explanation** |
| The use of too many words to convey one idea can muddle the message and divert the reader’s attention. Therefore, in writing, especially academic writing, ideas need to be conveyed as concisely as possible. One way of doing this is to use concise alternatives to phrases. For example, the phrase “all over the world” can be replaced with the word “globally” or “worldwide.”  Concise alternatives can also lend a more formal tone to the sentence. For example, “gradually” is considered a more formal alternative to “little by little” and is preferred in academic writing.  Finally, where possible, a direct verb (action) should be used instead of using a noun and verb. For example, “segmentation of images was done” can be replaced with “images were segmented,” which is clearer and preferred in academic writing. |
| **Example** |
| In addition, HCV treatment was effective in reducing the risk of insulin resistance and diabetes  Changed to:  In addition, HCV treatment reduces the risk of insulin resistance and diabetes. |
